# Supplementary material for: A global assessment of marine heatwaves and their drivers
Source: Nat Commun. 2019 Jun 14;10:2624. doi: 10.1038/s41467-019-10206-z (PMC6570771; doi:10.1038/s41467-019-10206-z)
Supplement: Supplementary file 1 — Supplementary Information [file 41467_2019_10206_MOESM1_ESM.docx]

**SUPPLEMENTARY INFORMATION**

**A global assessment of marine heatwaves and their drivers**

Holbrook et al.

**Supplementary Note 1 | Introduction**

This supplementary document contains more detailed information on the marine heatwave (MHW) literature review that informs the synthesis, summary and confidence assessment presented in the main text. The search criteria are constrained by the qualitative MHW definition^74^ and by observations from 1950-2016 (Supplementary Table 1). We consider the local processes responsible for ocean temperature changes underpinning MHWs in the upper ocean as informed by the mixed layer temperature tendency budget (Supplementary Fig. 1).

The remainder of this document provides an extended literature review and synthesis around these extreme warm water events organized by geographic typology including the eastern and western boundary currents, tropical latitudes, and middle and high latitudes. Supplementary Table 1 summarizes the main climate modes and processes, with a confidence rating dependent on the literature (Supplementary Fig. 2). Supplementary Table 2 summarizes for each region the date of the recorded MHW, location, intensity, and associated references based on a quantitative analysis. Supplementary Table 3 outlines the selection criteria applied to identify the relevant marine heatwave literature. Supplementary Tables 4-20 provide quick reference to the relevant literature on events identified in the case study regions. Finally, Supplementary Figs. 3-15 show statistical analyses of the relationships between climate mode phase and MHW occurrences.

**Supplementary Note 2 | Eastern Boundary Currents**

**a. Benguela Current**

A warm event occurred during the austral summer of 2001, persisted from February to April, and was confined to the coastal region off West Africa^1^. It was initiated by the relaxation of trade winds in the western tropical Atlantic Ocean and westerly wind anomalies along the equator. This triggered an equatorial Kelvin wave which propagated eastward towards Africa and southward along the African coast toward the Benguela Frontal Zone^1^. Anomalous advection occurred from a strong poleward geostrophic current in the Angola–Benguela Current system. Meanwhile, the thermocline deepened and the upper-ocean was unseasonably warm. Cooling by passive heat fluxes via local wind stress led to the event decaying in late April. This event was remotely forced by anomalous winds rather than local processes.

During March 1995, a warm event occurred in the Benguela Current system and was concurrent with the annual propagation of the Angola–Benguela Frontal Zone when warm, saline waters intruded poleward^2,85^. Warming extended from 24ºS to 5ºS and 300 km offshore of West Africa. A maximum temperature anomaly of 8ºC and subsurface warming to depths of 30–50 meters were observed and associated with a deepening of the thermocline^2^. The 1995 warm event was related to a concurrent Benguela Niño through the relaxation of easterly trade winds in the western equatorial Atlantic. An eastward propagating equatorial Kelvin wave bifurcated at the African coast as poleward propagating coastal Kelvin waves in both hemispheres causing warm water to advect across large geographic distances. The 1995 event was not associated with local winds.

In summary, the drivers of the 1995 and 2001 Benguela warm events originated in the tropical Atlantic through anomalous wind patterns. Anomalous westward winds triggered oceanic and coastal Kelvin waves, which enhanced geostrophic flow along the west coast of Africa and led to positive sea surface temperature (SST) anomalies during the late austral summer. These events are collectively referred to as Benguela Niño and represent an intensification of the annual cycle^3^. The seasonal timing of the extreme events (i.e. 1984, 1995, 1999) suggest phase locking of Benguela Niño to the late austral summer. The Benguela Niño is driven by climate variations that originate in the southeast Atlantic and the phenomenon typically occurs once every 10-15 years (i.e. 1934, 1949, 1963, 1984, 1995, 2001)^1–3^.

In addition to the Benguela Niño, the Angola–Benguela Frontal Zone is related to wind forcing and a seasonal Kelvin wave that originates from equatorial currents that trigger downwelling waves along the African coast. Warm saline waters protrude poleward causing anomalous advection and surface heat-fluxes^86^. The associated warm events are seasonally phase-locked to the late austral summer when northward wind stress and upwelling along the Namibian and Angolan coasts are significantly reduced.

**b. Leeuwin Current**

In late February 2011, a major and unprecedented MHW occurred off Western Australia, with peak temperatures exceeding 5ºC (4 standard deviations) above the 2000-2009 climatology^6^. The warm event, known as a ‘Ningaloo Niño’, extended from Ningaloo Reef to Cape Leeuwin and seaward out to 200 km. This event was caused by anomalous air-sea heat flux into the ocean and the poleward advection of warm, low saline tropical waters by the Leeuwin Current^5–7,87,59^. While the near-shore, wind-driven Capes Current is typically equatorward and upwelling favorable during summer, the current was suppressed as poleward flows dominated the shelf region^5^. The poleward advection of warm water contributed two-thirds of the warming, while positive air-sea heat fluxes into the ocean accounted for approximately one-third^5^.

The poleward strengthening of the Leeuwin Current was driven by prolonged easterly winds in the equatorial Pacific Ocean and reduced southerly winds typically opposing the alongshore current^7^. The equatorial Pacific easterly wind anomalies were related to the El Niño-Southern Oscillation (ENSO) behavior in the Pacific Ocean^75^ and occurred during a near-record La Niña. High sea level anomalies in the western Pacific Ocean propagated through the Indonesian Seas and led to high coastal sea level anomalies along Western Australia^4,75^. These high steric height anomalies forced a stronger than normal, poleward flowing Leeuwin Current. In addition, northerly wind anomalies associated with a low sea level pressure system intensified the Leeuwin Current and reduced the turbulent heat loss^6^. Anomalous air-sea heat fluxes reinforced the surface warming rather than damped SST anomalies^6^. After the peak warming along the coast occurred, positive sea level and SST anomalies propagated offshore due to eddies, allowing ocean temperatures to cool from air-sea heat fluxes^5^.

In the following two summers (2012, 2013), anomalously warm SSTs were recorded off Western Australia^88^. These events occurred during an increase in recorded Ningaloo Niño conditions since the early 1990s^40^. This increase has been attributed to the change to a negative phase of the Interdecadal Pacific Oscillation (IPO) and enhanced ENSO variance since the 1970s^40^. Studies on other Ningaloo Niño events indicated the importance of interannual variability related to ENSO^6^ and by local alongshore wind anomalies off the northwest Australian shelf^89^. The cyclonic atmospheric circulation anomaly reduced the prevailing alongshore southerly winds resulting in surface warming due to reduced latent heat loss and increased poleward transport of the Leeuwin Current. Coupling between the along-shore wind and coastal SST has been shown to amplify Ningaloo Niño^76^.

**c. California Current and off Baja California**

The California Current forms the eastern boundary of the North Pacific subtropical gyre and flows equatorward along the California coast. Hydrographic data from the California Cooperative Oceanic Fisheries Investigations (CalCOFI) survey show that an opposing poleward surface current (i.e. Davidson Current) over the continental slope occurs seasonally from October to February, while nearshore wind reversal can occur during late spring through summer when equatorward winds increase^90,91^. These seasonal surface flow patterns are most obvious along the central California coast. Anomalous warming events arise if there is an unusual poleward current during the summer season. Variability is driven by changes in meridional wind stress, alongshore pressure gradients, and wind stress curl^11^. A reduction in upwelling-favorable winds can weaken (and often reverse) equatorward currents^92^. Wind relaxation can lead to anomalous warming over the inner continental shelf by poleward advection of warm water and the displacement of cold upwelled water offshore^11,92^.

During June/July 1981 and in July 1984, a two to three week period of calm winds reversed the usual southward flowing surface current off the California coast^91^. These events were associated with anomalously warm surface water when upwelling-favorable winds relaxed. A model study^93^ showed that temperature variability within the upper 30 m of the water column off northern California was primarily driven by near-surface heat flux and poleward advection of heat by surface currents.

Off Baja California and Mexico, there is a small-scale, coupled air-sea phenomenon known as the California Niño/Niña and is associated with warm/cool SST anomalies along the coast. When equatorward winds relax, coastal upwelling is suppressed and warm SST anomalies develop in the ocean mixed-layer^12^. Positive SST anomalies induce heat flux out of the ocean, thereby warming the atmosphere and decreasing atmospheric pressure. The atmospheric response to the warm SST anomalies maintains the poleward flow of anomalous surface currents that are unfavorable to upwelling conditions^12^. During boreal summer, a possible coastal Bjerknes feedback can explain approximately half of the variability during California Niño/Niña, which is considered an independent phenomenon from ENSO.

During the 1997/98 El Niño, an anomalous large-scale cyclonic flow in the North Pacific Subtropical Gyre east of 125ºW caused warm and salty waters from the Central Pacific Gyre to shift towards Baja California. This created an unusual warm pool of SST anomalies similar in magnitude to those in the eastern equatorial Pacific during the peak of the event. Waters were 8.7ºC warmer and 0.8 saltier between October 1997 and January 1998^10^.

The causes and consequences of warm events in the California Current off San Diego during the boreal winters (DJF) of 1957/58, 1972/73, 1976/77, and 1982/83 have been explored^8^. Anomalous surface warming deepened the thermocline by 10-40 meters during these events, thereby enhancing sea level at tide gauges (6-16 cm) in San Diego. Persistent SST anomalies during 1957/58 and 1982/83 near the Scripps Pier ranged from 1.2-1.7ºC above average^8^. It was proposed that warming is caused by the depression of the thermocline from the poleward propagation of coastally trapped waves following El Niño events in the tropics, and by anomalous southwesterly winds that advect warm surface waters onshore^8^. Documented warming in the California Current co-occurred during tropical El Niño events during the boreal winters of 1972/73, 1976-78, and 1982/83^9^. Recent research has shown that atmospheric dynamics and tropical-extratropical teleconnections were also important for the spatial evolution and persistence of the more recent SST anomalies in 2015 ^72^. During these warm events, poleward range extensions of marine species were documented due to strengthening of the seasonal undercurrent that introduced warm tropical water to the region, making conditions habitable for southern species of plankton and fish (e.g. barracuda and white sea bass)^8^. A reduction in upwelling during these events decreased nutrients, which had negative consequences on algal growth, zooplankton productivity, and marine predators.

**d. Iberian/Canary**

McLain et al. (1985)^8^ reported weak and local positive anomalies during 1978, 1979/80, 1981/82, 1983/84 in the Canary upwelling region but did not provide details on individual warm events. Warm events occurred in the region of high seasonal SST variability in the vicinity of the frontal zone between 10ºN and 20ºN near Cape Vert and Cape Blanc.

**e. Humboldt/Peru**

During the 1982/83 El Niño, warm SST anomalies originating from the equatorial Pacific Ocean propagated southward along the South American coast. Coastal Kelvin waves excited by wind reversals in the central and western Pacific Ocean propagated the warm ENSO signal poleward away from the equator at the eastern boundary – identified as the propagation of positive sea level height anomalies along the coast and depression of the thermocline^14,16^. SSTs along the coast increased 6ºC above climatological norms in June 1983^14^ and 8-9ºC in December 1997 and March 1998 during another extreme El Niño event^18^. Smith (1983)^14^ reported an intensification of the Peru Coastal Undercurrent during the 1976 and 1982 El Niño events. The stronger poleward flow in the Southern Hemisphere caused anomalously warm water along the coast of South America. This elevated the intensity of the coastal undercurrent that supplied warmer-than-average water, which led to positive SSTs locally. During the 1997/98 El Niño, elevated Ekman pumping (downwelling) along the coast near 15ºS deepened the coastal thermocline in response to weakened southeasterly trade winds, which elevated SST along the coast^17^. Changes in coastal upwelling during the 1982/83 El Niño influenced biological productivity along the Peruvian coast^15^.

**Supplementary Note 3 | Western Boundary Currents and Extension regions**

Based on our literature survey, we have not identified any specific MHW events located in the core of the subtropical western boundary currents, although there have been events identified in the western boundary current extension regions. There is literature, however, that describes factors affecting upper ocean temperatures in these regions (summarized in Supplementary Table 1).

**a. Gulf Stream**

The latitudinal extent of the Gulf Stream is influenced by variations in large-scale atmospheric circulation known as the North Atlantic Oscillation (NAO) through changes in sea level pressure and surface wind stress. A northward displacement occurs approximately 11-18 months after the positive peak in the NAO. Warming north of the Gulf Stream occurs just before the northward shift in the Gulf Stream position and is part of the large-scale tripole pattern of SST anomalies in the North Atlantic caused by NAO forcing^19^. Positive SST anomalies emerge primarily from mixed layer deepening and surface heat exchange, and secondarily by horizontal SST advection^19^.

**b. Kuroshio-Oyashio**

Temperature fluctuations in the Kuroshio/Oyashio region are mostly associated with meanders of the boundary current and their extensions, and mesoscale eddies^94^. Temperature variability in the region influences pelagic fisheries and regional climate^95,96^.

**c. Brazil-Malvinas Confluence**

In the southwestern Atlantic Ocean between 35°S and 45°S, the warm, salty, and poleward flowing Brazil Current meets with the cold, fresh, and equatorward flowing Malvinas Current, forming the Brazil-Malvinas Confluence region^22,23,97^. There are strong variations in air-sea heat fluxes with heat losted and gained to the north and south, respectively, of the confluence region^22^. The Brazil Current separates from the coast, forming a recirculation and the Brazil Current Front, and the Malvinas Current returns southward and then offshore into the Subantarctic Front^23^. While there are small SST variations onshore of the Brazil Current, there are larger cross-front SST gradients offshore ^23^. In these regions, the seasonal frontal intensity is controlled by a number of factors including solar radiation, seasonal cooling, wind-driven mixing, and horizontal advection ^23^. Near the shelf-edge in the confluence region, large SST variations can occur through shifts in the location of the separation latitude, and the local SST depends on the local dominance of the Brazil or Malvinas Current^97^. Offshore, non-seasonal variability occurs through the eddy-rich field and meanders which can cause large local changes in SST^97^. Over interannual timescales, SSTs are anomalously warm in the Brazil Current during El Niño events, whereas they are anomalously warm in the Malvinas Current during La Niña events^98^.

**d. Agulhas Current**

The core region of the Agulhas Current extends south of the Mozambique Channel towards the southern tip of South Africa. It is the strongest western boundary current in the Southern Hemisphere and plays an important role in transporting heat and salinity from the equator towards the southern Indian and Atlantic Oceans. The temperature and speed of the Agulhas Current is influenced by interannual oscillations in the climate system, associated with ENSO, that propagate temperature anomalies through changes in wind stress and the excitation of oceanic Rossby waves^99^. Long-term warming trends in Agulhas Current SST have also been observed since 1980 caused by increased wind stress curl over the Indian Ocean that drives lateral advection of warm surface currents^25,26^. The increase in transport has been found to correspond to increased heat and salt flux into the South Atlantic Ocean.

**e. Agulhas Retroflection**

Shannon et al (1990)^77^ describe an anomalous warming to the south of Africa in late 1985 that migrated to the Benguela regions of the southeast Atlantic, resulting in warm conditions for much of 1986. They suggest an anmalous incursion of the Agulhas Retroflection into the southeast Atlantic from late 1985, drawing warm water from the Agulhas Current and thereby brining anomalously warm conditions to the southeast Atlantic (the warmast on record in certain locations since the start of the 20^th^ century). An Agulhas ring subsequently formed which remained attached to the retroflection for some months before moving northwards and westwards. A lack of observational data precluded drawing any firm conclusions regarding the mechanisms driving these changes. However, supported by pervious modelling studies^100^, they suggest that a southward deplacement of the zero wind stress curl reduced the volume transport of Aghulas water but allowed more of the warm water to penetrate into the Atlantic around the Cape of Good Hope.

**f. East Australian Current**

The core of the East Australian Current (EAC) represents the western Pacific arm of the South Pacific Subtropical Gyre which flows poleward (southward) along the east coast of Australia and comprises interactions between the mesoscale field and complex topography^101^. The core of the EAC extends from around 18^o^S, where the incoming South Equatorial Current bifurcates off northeast Australia and separates from the coast at around 32-33^o^S as the Tasman Front^102^. The EAC is energetic and regularly sheds eddies^103^ due to intrinsic instabilities^103,104^ and contributions from local wind stress variability at time scales <56 days^105^. Additionally, multi-year to decadal scale variations in the EAC appear to be due to important contributions from remote sources^28,106^. At coastal sites adjacent to the EAC, MHWs have been detected at the surface and with depth, and are found to be modulated by downwelling favorable winds^107^.

**g. East Australian Current Extension/Tasman Sea**

The Tasman Sea is supplied by warm water in the East Australian Current (EAC) Extension from the north. Variations in the strength of the EAC can affect temperature extremes through changes in the Tasman Sea eddy field in the EAC Extension region. Under future climate scenarios, warm anticyclonic eddies from the EAC are expected to become more abundant, longer lasting, and more stable^108^.

In September 2015, an unprecedented MHW commenced in the western Tasman Sea (off Tasmania) that lasted for 251 days^30^. The event resulted in mean SST anomalies of ~+3^o^C above climatological norms for the region and was primarily due to anomalously strong advection in the EAC Extension, composed mainly of warm-core eddies. The study also demonstrated that this MHW event was significantly more likely to have occurred due to anthropogenic climate change than natural variations alone.

**Supplementary Note 4 | Tropical Latitudes**

**a. Great Barrier Reef**

Along the Great Barrier Reef (GBR), extreme warming of marine waters have had detrimental impacts on coral reefs, notably through coral bleaching. Coral bleaching occurs as a stress response when the SST exceeds a temperature threshold – typically considered with respect to the monthly maximum SST – for an extended period of time^109^. Multiple factors are thought to affect the characteristics of warming events on the GBR, including the effects of global warming. SST records over the GBR have indicated a warming trend present throughout the 20^th^ Century^109^. From 1903 to 1999, the average annual SST over the GBR has warmed by ~0.6°C, and projected temperature rises may impact coral reef ecosystems by thermal stress^109^.

ENSO is a major source of interannual climate variability, and during El Niño (La Niña), the equatorial eastern Pacific is unusually warm (cold). During the major 1997/98 El Niño event, atypically warm SST over the GBR persisted for many days causing severe bleaching. In early February 1998, conditions were favorable for surface heating, with the alignment of low winds, neap tides, and clear skies for repeated periods of several days^35^. Weakened winds, i.e. doldrums conditions, can lead to reduced vertical mixing, enhance settlement of particulate matter which can increase light penetration, and intensify surface heating^33^. During February 1998, the surface waters continued to warm until vertical mixing with the cooler bottom waters occurred by wind and/or tidal processes. In the Central GBR, this warming was followed by a strengthening of the southeasterly winds, leading to a northward coastal current that advected the warm water northward. In the Southern GBR, cloud cover and a mismatch between low wind speeds and neap tides helped reduce surface warming compared to the central and northern GBR^35^.

During austral summer of 2001/02, an even more severe bleaching event occurred in the GBR in terms of numbers of reefs affected and bleaching intensity. Factors include weakened winds with reduced evaporation and higher solar radiation^36,110^. This event was also concurrent with an El Niño, albeit considerably weaker than in 1998. Both 1998 and 2002 periods led to more coral bleaching along inshore reefs than offshore reefs^31^, which may be due to greater temperature extremes in the shallow inshore waters during summer^111^. Both events were associated with an increase in net surface heat flux.

Most recently, a major coral bleaching event occurred during austral summer 2015/16, which predominantly impacted the northern GBR^78,79,81^. The spatial patterns in bleaching followed a similar pattern for thermal stresses^78^, with northern GBR having SSTs that were the highest on record^79^. Warming across the northern GBR has been associated with advection^112^ and air-sea heat flux anomalies^81^. Meanwhile, in the southern GBR, the passage of a cyclone was associated with cooling and reduced thermal stresses on corals there^113^. This time period coincided with a major El Niño^79^, and anthropogenic warming increased the likelihood of this extreme warming event^80^.

**b. Seychelles Islands**

Like many other regions globally, the western Indian Ocean was impacted by warming in 1998, with extensive coral bleaching^115,116^. Positive SST anomalies above climatological norms occurred over a widespread region. The inner Seychelles, which is a shallow 7-9 m continental shelf basin, had one of the largest coral losses during this event^115^. In the southern Seychelles (6-10°S, 45-54°E), the warm event lasted for more than five months, with SST anomalies at different sites ranging from +1-2^o^C above normal maximum temperatures^39^. During the 1997/98 El Niño, easterly wind anomalies along the equator of the Indian Ocean weakened the trade winds to the south. The equatorial region warmed due to less latent heat release from the weakened winds, and Kelvin and westward propagating Rossby waves facilitated the basin-wide spread of the anomalous warming^39^.

Analyses of HadISST time series from 1951-2002 reveals an underlying warming of the mean and maximum SST in east African coastal waters by +0.01°C/year^117^. During this time-period, the mean SSTs of these waters were the highest during El Niño years 1982/83, 1987/88, and 1997/98. The 1997/98 event had both the highest mean and maximum SST and persisted the longest. Compared to non-ENSO years, ENSO years tend to show greater mean SST off the coast of east Africa. Warmest SST anomalies tend to be associated with ENSO, IOD, and ENSO-IOD events. The 1997/98 event had co-occurring ENSO and IOD events^118^.

**c. Galapagos Islands**

Much of the warm event research around the Galapagos Islands in the eastern equatorial Pacific Ocean (EEP) has been motivated by biological studies on coral bleaching^41,46^. Observations of coral bleaching near the Galapagos have coincided with strong El Niño events during 1983, 1987 and 1992, which caused extreme and prolonged SST anomalies^41^. Interannual variability of ocean temperatures in the EEP is dominated by ENSO.

Intense, long duration warm events in the EEP are often related to El Niño events. Several studies have investigated the processes affecting surface temperatures (e.g. ^119–123^). Changes in the vertical advection of heat (in particular the mean advection of anomalous vertical temperature gradients) are thought to be the primary driver of ENSO-related eastern equatorial SST anomalies. The change in thermocline depth associated with the temperature gradient change arises primarily from remote wind changes that generate eastward propagating downwelling equatorial Kelvin waves. However other factors, including local winds that alter the mean upwelling strength and the zonal advection of temperature, are also important in enhancing SST anomalies. Factors such as air-sea heat fluxes and mixing by tropical instability waves act to damp the SST anomalies.

**d. Bay of Bengal**

Marine heatwave events were observed in the Andaman Sea of the Bay of Bengal in 1998, 2002, 2005, and 2010. The April-May 2010 event was the strongest event in the record, causing severe coral bleaching in the region^125^. Most of the events occurred during or after strong El Niño events in the Pacific, which often induced Indian Ocean basin warming and negative Indian Ocean Dipole after the El Niño events. El Niño also induced warming events across the Bay of Bengal along the Indian and Sri Lanka coasts^126^, likely associated with weakened South Asian summer monsoon. Intraseasonal Oscillations (ISO) of the South Asian summer monsoon drove SST variability of up to 2°C in the Bay of Bengal, due to ISO induced net surface heat flux variability of up to 200 Wm^-2^ ^43^.

**e. Caribbean Sea**

The literature on Caribbean Sea warming events is limited to those reporting on coral bleaching, where the causes and mechanisms of warming are not well documented or known. Most Caribbean corals will expel their zooxanthellae (photosynthetic algae) once ocean temperatures exceed a threshold and this causes the corals to appear bleached. Extreme temperatures may also cause widespread mortality of reef building coral colonies and undermine the health of the reef ecosystem.

A major coral bleaching event in the Caribbean Sea occurred during the 1982/83 El Niño where hotspots exceeded 32ºC^46^. Although the locations of these warm pools were not explicitly stated in the literature, the authors deduced that warm anomalies were caused by a reduction in cloud cover and atmospheric teleconnections that stemmed from the eastern tropical Pacific Ocean^46^.

In 1987, the waters off the Caribbean, including Jamaica, were elevated from late July to March 1988^47^. The SSTs rose early and persisted for 9 months through the warm season, causing widespread coral bleaching. Jamaican reefs also experienced bleaching in 1989 when SSTs reached upwards of 30ºC as early as August^47^.

**Supplementary Note 5 | Middle and High Latitudes**

**a. Mediterranean Sea**

The Mediterranean Sea is a semi-enclosed sea where neighboring land conditions and atmospheric anomalies have a large effect on water temperatures. An atmospheric blocking system over Western Europe during the boreal summer of 2003 set-up a persistent anticyclone centred over northern France that was unprecedented at the time^48,50^. Reduced wind stress and turbulent heat loss passively increased SST in the Mediterranean Sea^50,52,54^. Anomalous radiative heat fluxes were associated with the intensification and northward displacement of the subtropical Azores High^50^.

Surface air temperatures during this event exceeded +3-6ºC above seasonal norms^51,52,54^. Warm, low-level air advected across continental Europe rather than the Atlantic. SST rose rapidly during late April to early May, and persisted through August 2003^50^. SST increased to +3-4ºC above seasonal mean conditions in the Mediterranean Sea, including the Gulf of Lions, Ligurian, Tyrrhenian, Northern Ionian, and Adriatic Seas^49^. The water column was well stratified during the heatwave due to little or no wind mixing^51,52^. Anomalies were confined to the upper surface layer^51,52,54^. The warm event ended abruptly in late August to early September when strong westerly winds cooled air temperatures and induced wind-driven turbulent mixing^54^.

Possible teleconnections driving the 2003 warm event in the Mediterranean Sea included a northward shift and intensification of the Intertropical Convergence Zone (ITCZ) over West Africa and Rossby wave signals from tropical America. Decadal fluctuations in North Atlantic SSTs and the thermohaline circulation are known to influence European weather over long timescales. During 2003, the Atlantic Multi-decadal Oscillation (AMO) index was positive and associated with elevated air temperatures and reduced wind stress over western Europe^52,127^.

A similar, albeit not as extreme, warm event occurred during the summer of 1999 in the Mediterranean Sea. As with 2003, overlying air temperatures in 1999 were well above normal. However, the water column was much more stable^51^. Consequently, the thermocline deepened by 30-40 metres and temperatures rose by 2-3ºC above the thermocline^53^.

The 1999 and 2003 warm events in the Mediterranean Sea bear physical resemblances in their origin and evolution. The MHWs in the Mediterranean Sea were atmospherically driven and arose from anomalous radiative heat fluxes and positive surface air temperature anomalies. Variable winds played a role in determining whether the SST anomalies were confined above the thermocline. In the case of the 1999 event, wind-driven mixing extended temperature anomalies to depths of 40 metres, whereas during the 2003 event virtually no winds led to isolated warming in the upper 20 meters of the water column. There is agreement from the literature suggesting that the strength and position of the Azores High had a large influence on the atmospheric conditions driving warm events in this region. However, there is limited evidence that the AMO was an important forcing mechanism. It is expected that the Mediterranean region will become warmer and drier in the 21^st^ century and that Mediterranean Sea warm events will become more frequent because of climate change^128^.

**b. Bering Sea**

The Bering Sea, situated in the far North Pacific between Alaska and Russia, is a deep marginal sea and one of the most productive marine ecosystems in the world. Variable winds from the Aleutian Low and large-scale atmospheric teleconnections from the tropical Pacific impose sudden changes in oceanographic conditions in this region^129^.

During the boreal summer of 1997, positive SST anomalies occurred over much of the eastern shelf of the Bering Sea and extended into the Gulf of Alaska^59,60^. SST anomalies peaked at +3ºC above average in June, but anomalous conditions were observed from the beginning of April through to August^59,61^. Warm water anomalies were confined to the shallow mixed layer (<20 metres), below which cold water anomalies were present. Strong stratification and a shallow mixed layer occurred along the shelf due to a shift in wind-driven frontal zones and vertical density gradients^57,130^.

This warm event co-occurred with one of the strongest El Niño events on record. Tropical deep convection, indicated by low outgoing longwave radiation over anomalous SSTs associated with El Niño, caused a deeper than normal Aleutian Low^55^. Poleward propagating atmospheric Rossby waves transmitted anomalies from the tropics to mid-latitudes. Warm SST anomalies extended from the equator along the west coast of North America, across the Gulf of Alaska, and into the Bering Sea^59^. However, there were no indications of a warm oceanic anomaly propagating from the equator^60,61^.

During May 1997, a blocking ridge of high pressure centred over the Gulf of Alaska prevented synoptic weather patterns from moving across the Bering Sea^57,59^. Unusually clear skies, warm air temperatures, and low relative humidity induced positive heat flux anomalies into the ocean, which rapidly warmed the shallow mixed layer. SST anomalies were +4ºC above climatological means within the shallow mixed layer^57^. Calm winds associated with the ridge of high pressure reduced typical wind-driven turbulent mixing. The warm event concluded with a strong Aleutian Low and the passage of frequent storms in November 1997^57^.

An additional multi-year warming event in the Bering Sea occurred during 2000-2005. Mean air temperatures were approximately +2ºC above the climatology and the eastern Bering Sea reached thresholds up to +3ºC warmer than usual. Although wind-driven warm water advection helped to sustain this event, atmospheric conditions associated with the Aleutian Low were primarily responsible^58^. Atmospheric conditions during this period were similar to those during the 1997 warm event.

Atmospheric circulation in the North Pacific is partially associated with ENSO on interannual timescales. In the late 1970s, the PDO underwent a phase shift resulting in a stronger Aleutian Low (defined by the 700mb geopotential height)^130^. The 1997 Bering Sea warm event was atmospherically driven due to changes in wind-driven frontal zones, a reduction in synoptic storms, reduction in cloud cover, and above average air temperatures. A similar warming from 2000-2005 may have been the result of the mid-1970s shift in global climate events related to ENSO^58^. An atmospheric decadal trend favoring a deeper Aleutian Low, ENSO atmospheric teleconnections, and strong synoptic weather patterns related to the blocking high pressure ridge, conspired to create anomalous heating in the shallow mixed layer in the eastern Bering Sea^57,59^. More work is needed to confirm if the interannual and intraseasonal drivers of these events act alone or whether they are related to one another.

**c. Northwest Atlantic Ocean**

The northwest Atlantic Ocean extends from the cold subpolar waters off Atlantic Canada and New England to temperate waters along the mid-Atlantic bight. Some of the most lucrative fisheries (e.g. lobster, cod, salmon and tuna) rely on the productive marine ecosystems within this region^65^. These fisheries were disrupted during a large-scale warm event during the boreal summer of 2012; however, precursors of this event were in place months before the oceanic warming was realized.

The peak of the northwest Atlantic warm event occurred during March and May of 2012, although significant SST anomalies reaching +1-3ºC above the 1982-2011 climatology persisted through late August^63,65^. The average anomaly during the event was +2ºC, or 3.5 standard deviations above normal^65^. Although most of the warming was concentrated towards the continental shelf and Gulf of Maine, the warm event covered an area from Cape Hatteras to Iceland and up into the Labrador Sea. Unusually warm conditions extended throughout the water column and an increase in bottom temperature was also observed^63,64^.

Chen et al.^64^ found evidence that the northwest Atlantic warm event originated from anomalous atmospheric conditions during the late autumn to early winter of 2011/12. A meridional shift in the jet stream position over North America led to reduced latent and sensible heat fluxes from the ocean during the winter preceding the event. The northward displacement of the jet stream led to the stabilisation of an atmospheric blocking pattern of high pressure characteristic of a reduction in overall wind speeds, high air humidity, and surface air temperatures. The intraseasonal oscillation of the jet stream in early 2012 (caused by mountain torques) prevented typical wintertime cooling in surface waters, and therefore predisposed ocean surface conditions to warming early in the year. Atmospheric blocking caused warm air to stabilize and persist throughout the summer season and induced positive heat flux anomalies into the ocean that stratified the water column and elevated SSTs.

**d. Northeast Pacific Ocean**

An unusually warm region (1.5x10^6^km^2^) in the northeast Pacific centred at 41-45ºN, 145-150ºW developed during the boreal winter of 2013/14. This event was nicknamed “the Blob” by Washington State’s Climatologist, Nicholas Bond and received mass media attention for its impacts on North American winter weather and northeast Pacific marine ecosystems. Among other impacts, the warm conditions were associated with a coastwide toxic algal bloom that affected shellfish and finfish fisheries and led to the strandings of several marine mammals^131^.

A persistent atmospheric ridge of high pressure stalled over the west coast of North America, Alaska, and high-latitude North Pacific Ocean^68,69^. Higher than normal sea level pressure during October 2013 to January 2014 was unprecedented since the 1980s and led to lower rates of seasonal heat loss from the ocean to the atmosphere^68,70,71^. Weaker winds induced weaker than normal Ekman transport of cold water from the north and reduced turbulent heat flux across the mixed layer. Zonal winds between October and January were the weakest on record^71^. The timing of maximum SST anomalies differed regionally, with coastal anomalies in the California Current Upwelling System preceding the ones offshore^132^. A mixed layer temperature budget for the northeast Pacific Ocean showed that anomalous temperatures were caused by unseasonably weak advection of cold water from the north and a reduction in surface heat loss^68^. SST anomalies peaked in February and were more than +2.5ºC (3 standard deviations) above climatological norms in the upper 100 meters of the water column^68^. Meanwhile, Lee et al.^69^ found that SST anomalies exceeded their 95^th^ percentile and Whitney^71^ showed that SST was +3.5ºC above average in January 2014. Most recently, research has shown that atmospheric dynamics and tropical-extratropical teleconnections were important for the spatial evolution and persistence of the SST anomalies in 2015^72^.

The anomalous warm event in the northeast Pacific Ocean was associated with the third mode of SST variability^70^, which is characteristic of high pressure in the northeast Pacific. From mid-2013 through January 2014 this mode was two standard deviations above normal. Additional forcing came from warm SSTs in the western tropical Pacific that had persisted since the winter of 2013. These anomalies disrupted the atmospheric circulation over the extra-tropics through the excitement of atmospheric Rossby waves^69,70^. There is also some indication that the Northeast Pacific warm event was associated with the North Pacific Oscillation^69^.

Low levels of sea ice in the Arctic and positive SST anomalies in the tropical Pacific Ocean modulated anomalous atmospheric circulation over the warm event leading to extreme and persistent warming in the surface waters during the boreal winter of 2013-2014^69,70^.

**e. South Central Pacific Ocean**

In 2009, a warming event approximately the size of the United States developed in the South-Central Pacific in the mid-latitudes. Anomalies developed in September 2009, rapidly grew by November and peaked in December at +2ºC, or five times the standard deviation above climatological norms^73^. Warming was confined to the mixed-layer depth (50 meters) during the austral spring and summer. By January the event started to decay and was completely gone by April. Anomalous warming in the Bellingshausen Sea also reached a three-decade high and was related to the South Pacific atmospheric heatwave through the diversion of warm mid-latitude air towards Western Antarctica^73^.

The South Pacific MHW co-occurred with the mature phase of the 2009/10 Central Pacific El Niño. The MHW was +0.5ºC warmer than ENSO anomalies in the tropical Pacific, and the 2009/10 Central Pacific El Niño was 60% more intense than all other El Niño events over the past three decades, setting a record for Niño4-region anomalies^73^. There is strong evidence that the Central Pacific El Niño played a significant role in the South Pacific MHW. The Southern Annular Mode (SAM) was not particularly unusual between October and December 2009, and so SAM most likely did not play a role in the formation of the persistent anticyclone.

Atmospheric pressure anomalies associated with ENSO typically travel to the South Pacific via atmospheric Rossby waves (e.g. Pacific-South American Mode) and through changes in the mid-latitude meridional circulation. Clear evidence of this mechanism was evident during the 2009/10 event^73^ shown by an intense and persistent atmospheric anticyclone, which blocked atmospheric flow, decreased wind speed, and reduced synoptic storms. This diverted the circumpolar westerlies and warm air poleward. Low wind speeds and an easterly wind anomaly reduced latent and sensible heat fluxes and increased SST^73^. The easterly wind anomalies forced an anomalous southward meridional Ekman flow that weakened the normal northward advection of cold water from the south.

The coupling between the wind anomalies and oceanic currents enhanced warming in the South Pacific during the warm event. Surface heat fluxes and ocean advection processes played equal roles and each accounted for approximately 40% of the warming event^73^. Weak turbulent mixing may have explained the remainder of the anomalous warming.

**Supplementary References**

1. Rouault, M., Illig, S., Bartholomae, C., Reason, C. J. C. & Bentamy, A. Propagation and origin of warm anomalies in the Angola Benguela upwelling system in 2001. *J. Mar. Syst.* **68**, 473–488 (2007).

2. Gammelsrød, T., Bartholomae, C. H., Boyer, D. C., Filipe, V. L. L. & O’Toole, M. J. Intrusion of warm surface water along the Angolan-Namibian coast in February–March 1995: the 1995 Benguela Nino. *South African J. Mar. Sci.* **19**, 41–56 (1998).

3. Shannon, L. V., Boyd, A. J., Brundrit, G. B. & Taunton-Clark, J. On the existence of an El Niño-type phenomenon in the Benguela System. *J. Mar. Res.* **44**, 495–520 (1986).

4. Caputi, N., Jackson, G. & Pearce, A. *The marine heat wave off Western Australia during the summer of 2010/11 – 2 years on Management implications of climate change effects on fisheries in WA: an example of an extreme event*. *Fisheries Research Report* (2014).

5. Benthuysen, J., Feng, M. & Zhong, L. Spatial patterns of warming off Western Australia during the 2011 Ningaloo Niño : Quantifying impacts of remote and local forcing. *Cont. Shelf Res.* **91**, 232–246 (2014).

6. Feng, M., McPhaden, M. J., Xie, S. P. & Hafner, J. La Niña forces unprecedented Leeuwin Current warming in 2011. *Sci. Rep.* **3**, 1277 (2013).

7. Pearce, A. F. & Feng, M. The rise and fall of the ‘marine heat wave’ off Western Australia during the summer of 2010/2011. *J. Mar. Syst.* **111**–**112**, 139–156 (2013).

8. McLain, D. R., Brainard, R. E. & Norton, J. G. Anomalous warm events in eastern boundary current systems. *CalCOFI Rep.* **26**, 51–64 (1985).

9. Norton, J., McLain, D., Brainard, R. & Husby, D. The 1982–83 El Niño event off Baja and Alta California and its ocean climate context. in *El Niño North: Niño effects in the Eastern Subarctic and Pacific Ocean* 44–72 (1985).

10. Durazo, R. & Baumgartner, T. . R. Evolution of oceanographic conditions off Baja California : *Prog. Oceanogr.* **54**, 7–31 (2002).

11. Melton, C., Washburn, L. & Gotschalk, C. Wind relaxations and poleward flow events in a coastal upwelling system on the central California coast. *J. Geophys. Res. Ocean.* **114**, 1–18 (2009).

12. Yuan, C. & Yamagata, T. California Niño/Niña. *Sci. Rep.* **4**, 4801 (2014).

13. DeCastro, M., Gõmez-Gesteira, M., Costoya, X. & Santos, F. Upwelling influence on the number of extreme hot SST days along the Canary upwelling ecosystem. *J. Geophys. Res. Ocean.* **119**, 3029–3040 (2014).

14. Cane, M. A. Oceanographic Events During El Nino. *Science (80-. ).* **222**, 1189–1195 (1983).

15. Barber, R. T. & Chavez, F. P. Biological Consequences of El Niño. *Science (80-. ).* **222**, 1203–1210 (1983).

16. Smith, R. L. Peru coastal currents during el nino: 1976 and 1982. *Science (80-. ).* **221**, 1397–1399 (1983).

17. Halpern, D. Offshore Ekman transport and Ekman pumping off Peru during the 1997-1998 El Niño. *Geophys. Res. Lett.* **29**, 19-1-19–4 (2002).

18. McPhaden, M. J. Genesis and evolution of the 1997-98 El Niño. *Science* **283**, 950–954 (1999).

19. Frankignoul, C., de Coëtlogon, G., Joyce, T. M. & Dong, S. Gulf Stream variability and ocean–atmosphere interactions. *J. Phys. Oceanogr.* **31**, 3516–3529 (2001).

20. Kwon, Y. O. *et al.* Role of the gulf Stream and Kuroshio-Oyashio systems in large-scale atmosphere-ocean interaction: A review. *J. Clim.* **23**, 3249–3281 (2010).

21. Garcia, C. A. E., Sarma, Y. V. B., Mata, M. M. & Garcia, V. M. T. Chlorophyll variability and eddies in the Brazil-Malvinas Confluence region. *Deep. Res. Part II Top. Stud. Oceanogr.* **51**, 159–172 (2004).

22. Provost, C., Garcia, O. & Garçon, V. Analysis of satellite sea surface temperature time series in the Brazil-Malvinas Current Confluence region: Dominance of the annual and semiannual periods. *J. Geophys. Res.* **97**, 17841–17858 (1992).

23. Saraceno, M., Provost, C., Piola, A. R., Bava, J. & Gagliardini, A. Brazil Malvinas Frontal System as seen from 9 years of advanced very high resolution radiometer data. *J. Geophys. Res. C Ocean.* **109**, (2004).

24. Pezzi, L. P. *et al.* Ocean-atmosphere in situ observations at the Brazil-Malvinas Confluence region. *Geophys. Res. Lett.* **32**, 1–4 (2005).

25. Rouault, M., Penven, P. & Pohl, B. Warming in the Agulhas Current system since the 1980’s. *Geophys. Res. Lett.* **36**, L12602 (2009).

26. Zinke, J., Loveday, B. R., Reason, C. J. C., Dullo, W.-C. & Kroon, D. Madagascar corals track sea surface temperature variability in the Agulhas Current core region over the past 334 years. *Sci. Rep.* **4**, 4393 (2014).

27. Holbrook, N. J. & Bindoff, N. L. Interannual and decadal temperature variability in the southwest Pacific Ocean between 1955 and 1988. *J. Clim.* **10**, 1035–1049 (1997).

28. Holbrook, N. J., Goodwin, I. D., McGregor, S., Molina, E. & Power, S. B. ENSO to multi-decadal time scale changes in East Australian Current transports and Fort Denison sea level: Oceanic Rossby waves as the connecting mechanism. *Deep. Res. Part II Top. Stud. Oceanogr.* **58**, 547–558 (2011).

29. Malcolm, H. A., Davies, P. L., Jordan, A. & Smith, S. D. A. Variation in sea temperature and the East Australian Current in the Solitary Islands region between 2001-2008. *Deep. Res. Part II Top. Stud. Oceanogr.* **58**, 616–627 (2011).

30. Oliver, E. C. J. *et al.* The unprecedented 2015/16 Tasman Sea marine heatwave. *Nat. Commun.* **8**, (2017).

31. Berkelmans, R., De’ath, G., Kininmonth, S. & Skirving, W. J. A comparison of the 1998 and 2002 coral bleaching events on the Great Barrier Reef: Spatial correlation, patterns, and predictions. *Coral Reefs* **23**, 74–83 (2004).

32. Redondo-Rodriguez, A., Weeks, S. J., Berkelmans, R., Hoegh-Guldberg, O. & Lough, J. M. Climate variability of the Great Barrier Reef in relation to the tropical Pacific and El Niño-Southern Oscillation. *Mar. Freshw. Res.* **63**, 34–47 (2012).

33. Berkelmans, R., Weeks, S. J. & Steinberga, C. R. Upwelling linked to warm summers and bleaching on the Great Barrier Reef. *Limnol. Oceanogr.* **55**, 2634–2644 (2010).

34. McPhaden, M. J. Genesis and evolution of the 1997-98 El Nino. *Science* **283**, 950–954 (1999).

35. Skirving, W. & Guinotte, J. The sea surface temperature story on the great barrier reef during the coral bleaching event of 1998. in *Oceanographic Processes of Coral Reefs: Physical and Biological Links in the Great Barrier Reef* (ed. Wolanski, E.) 301–313 (CRC Press, 2000). doi:10.1201/9781420041675

36. Schiller, A., Ridgway, K. R., Steinberg, C. R. & Oke, P. R. Dynamics of three anomalous SST events in the Coral Sea. *Geophys. Res. Lett.* **36**, L06606 (2009).

37. Murtugudde, R., McCreary, J. P. & Busalacchi, A. J. Oceanic processes associated with anomalous events in the Indian Ocean with relevance to 1997–1998. *J. Geophys. Res.* **105**, 3295 (2000).

38. Webster, P. J., Moore, a M., Loschnigg, J. P. & Leben, R. R. Coupled ocean-atmosphere dynamics in the Indian Ocean during 1997-98. *Nature* **401**, 356–360 (1999).

39. Spencer, T., Teleki, K. A., Bradshaw, C. & Spalding, M. D. Coral bleaching in the Southern Seychelles during the 1997-1998 Indian Ocean warm event. *Mar. Pollut. Bull.* **40**, 569–586 (2000).

40. Yu, L. & Rienecker, M. M. Mechanisms for the Indian Ocean warming during the 1997–98 El Niño. *Geophys. Res. Lett.* **26**, 735 (1999).

41. Podestà, G. P. & Glynn, P. W. Galfipagos : Extreme temperatures causing coral bleaching. *J. Geophys. Res.* **102 NO. C7**, 15749–15759 (1997).

42. Podestà, G. P. & Glynn, P. W. The 1997-98 El Nino event in Panama and Galapagos: an update of thermal stress indices relative to coral bleaching. *Bull. Mar. Sci.* **69**, 43–59 (2001).

43. Sengupta, D. & Ravichandran, M. Oscillations of bay of Bengal sea surface temperature during the 1998 summer monsoon. *Geophys. Res. Lett.* **28**, 2033–2036 (2001).

44. Farukh, M. A., Chowdhury, M. J. R. & Basak, S. C. Sea Surface Temperature Anomaly in the Bay of Bengal in 2010. **5**, 77–80 (2012).

45. Jadhav, S. K. & Munot, A. A. Warming SST of Bay of Bengal and decrease in formation of cyclonic disturbances over the Indian region during southwest monsoon season. *Theor. Appl. Climatol.* **96**, 327–336 (2009).

46. Roberts, L. Coral Bleaching Threatens Atlantic Reefs: Unexplained changes are occurring in some of the most productive ecosystems on the planet, the Caribbean coral reefs. *Science* **238**, 1228–9 (1987).

47. Goreau, T. J. Coral Bleaching in Jamaica. *Nature* **343**, 417 (1990).

48. Grazzini, F., Ferranti, L., Lalaurette, F. & Vitart, F. The exceptional warm anomalies of summer 2003. *ECMWF Newsl.* **99**, 2–8 (2003).

49. Marullo, S. & Guarracino, M. Satellite observation of the 2003 thermal anomaly in the Mediterranean Sea. 48–53 (2003).

50. Black, E., Blackburn, M., Harrison, R. G., Hoskins, B. J. & Methven, J. Factors contributing to the summer 2003 European heatwave. *R. Meteorol. Soc.* **59**, 217–223 (2004).

51. Garrabou, J. *et al.* Mass mortality in Northwestern Mediterranean rocky benthic communities: Effects of the 2003 heat wave. *Glob. Chang. Biol.* **15**, 1090–1103 (2009).

52. Olita, A., Sorgente, R., Ribotti, A., Natale, S. & Gaberšek, S. Effects of the 2003 European heatwave on the Central Mediterranean Sea surface layer: a numerical simulation. *Ocean Sci. Discuss.* **3**, 85–125 (2006).

53. Romano, J.-C., Bensoussan, N., Younes, W. A. & Arlhac, D. Thermal anomaly in waters of the Gulf of Marseilles during the summer of 1999. A partial explanation of the mortality of certain fixed invertebrates? *C. R. Acad. Sci. III.* **323**, 415–27 (2000).

54. Sparnocchia, S., Schiano, M. E., Picco, P., Bozzano, R. & Cappelletti, A. The anomalous warming of summer 2003 in the surface layer of the Central Ligurian Sea (Western Mediterranean). *Ann. Geophys.* **24**, 443–452 (2006).

55. Stabeno, P. J. The status of the Bering Sea during the first 8 months of 1998. 6–8 (1998).

56. Yeh, S. W. S. Y. K. K. S. *et al.* Recent climate variation in the Bering and Chukchi Seas and its linkages to large-scale circulation in the Pacific. *Clim. Dyn.* **42**, 2423–2437 (2014).

57. Napp, J. M. & Hunt, G. L. Anomalous conditions in the south-eastern Bering Sea 1997: Linkages among climate, weather, ocean, and biology. *Fish. Oceanogr.* **10**, 61–68 (2001).

58. Overland, J. E., Wang, M., Wood, K. R., Percival, D. B. & Bond, N. A. Deep-Sea Research II Recent Bering Sea warm and cold events in a 95-year context. *Deep. Res. Part II* **65**–**70**, 6–13 (2012).

59. Overland, J. E., Bond, N. A. & Adams, J. M. North Pacific Atmospheric and SST anomalies in 1997: Links to ENSO? *Fish. Oceanogr.* **10**, 69–80 (2001).

60. Schumacher, J. D., Stabeno, P. J., Bond, N. A. & Napp, J. M. *Ecosystem Anomalies in the Eastern Bering Sea During 1997*. *North Pacific Anadromous Fish Commission, Technical Report* (1998).

61. Vance, T. *et al.* Aquamarine waters recorded for first time in eastern bering sea. *Eos, Trans. Am. Geophys. Union* **79**, 121–121 (1998).

62. Ding, Q. *et al.* Tropical forcing of the recent rapid Arctic warming in northeastern Canada and Greenland. *Nature* **509**, 209–12 (2014).

63. Friedland, K. Ecosystem Advisory for the Northeast Shelf Large Marine Ecosystem. *Advis. 2014, No. 1* (2012).

64. Chen, K., Gawarkiewicz, G. G., Lentz, S. J. & Bane, J. M. Diagnosing the warming of the Northeastern U.S. Coastal Ocean in 2012: A linkage between the atmospheric jet stream variability and ocean response. *J. Geophys. Res. Ocean.* **119**, 218–227 (2014).

65. Mills, K. E. *et al.* Fisheries Management in a Changing Climate Lessons from the 2012 ocean Heat Wave in the Northwest Atlantic. *Oceanography* **26**, 191–195 (2013).

66. Chen, K., Gawarkiewicz, G., Kwon, Y.-O. & Zhang, W. The role of atmospheric forcing versus ocean advection during the extreme warming of the Northeast U.S. continental shelf in 2012. *J. Geophys. Res. Ocean.* **120**, 4324–4339 (2015).

67. Scannell, H., Pershing, A., A., A. M., Thomas, A. C. & Mills, K. E. Frequency of marine heatwaves in the North Atlantic and North Pacific since 1950. *Geophys. Res. Lett.* **43**, 2069–2076 (2016).

68. Bond, N. A., Cronin, M. F., Freeland, H. & Mantua, N. Causes and impacts of the 2014 warm anomaly in the NE Pacific. *Geophys. Res. Lett.* **42**, 3414–3420 (2015).

69. Lee, M., Hong, C. & Hsu, H. Compounding effects of warm sea surface temperature and reduced sea ice on the extreme circulation over the extratropical North Pacific and North America during the 2013–2014 boreal winter. *Geophys. Res. Lett.* **42**, 1612–1618 (2015).

70. Hartmann, D. L. Hartmann-2015-Geophysical_Research_Letters. *Geophys. Res. Lett.* **42**, 1894–1902 (2015).

71. Whitney, F. A. Anomalous winter winds decrease 2014 transition zone productivity in the NE Pacific. *Geophys. Res. Lett.* **2014**, 428–431 (2015).

72. Di Lorenzo, E. & Mantua, N. Multi-year persistence of the 2014/15 North Pacific marine heatwave. *Nat. Clim. Chang.* **6**, 1042–1047 (2016).

73. Lee, T. *et al.* Record warming in the South Pacific and western Antarctica associated with the strong central-Pacific El Nio in 2009-10. *Geophys. Res. Lett.* **37**, 1–6 (2010).

74. Hobday, A. J. *et al.* A hierarchical approach to defining marine heatwaves. *Prog. Oceanogr.* **141**, 227–238 (2016).

75. Feng, M. *et al.* Decadal increase in Ningaloo Niño since the late 1990s. *Geophys. Res. Lett.* **42**, 104–112 (2015).

76. Kataoka, T., Tozuka, T., Behera, S. & Yamagata, T. On the Ningaloo Niño/Niña. *Clim. Dyn.* **43**, 1463–1482 (2014).

77. Shannon, L. V., Agenbag, J. J., Walker, N. D. & Lutjeharms, J. R. E. A major perturbation in the Agulhas retroflection area in 1986. *Deep Sea Res. Part A, Oceanogr. Res. Pap.* **37**, 493–512 (1990).

78. Hughes, T. P. *et al.* Global warming and recurrent mass bleaching of corals. *Nature* **543**, 373–378 (2017).

79. Great Berrier Reef Marine Park Authority 2017. *Final report : 2016 coral bleaching event on the Great Barrier Reef*.

80. King, A. D., Karoly, D. J. & Henley, B. J. Australian climate extremes at 1.5 ^o^C and 2^o^C of global warming. *Nat. Clim. Chang.* **7**, 412–418 (2017).

81. Benthuysen, J. A., Oliver, E. C. J., Feng, M. & Marshall, A. G. Extreme Marine Warming Across Tropical Australia During Austral Summer 2015–2016. in *Journal of Geophysical Research: Oceans* **123**, 1301–1326 (2018).

82. Romano, J.-C., Bensoussan, N., Younes, W. a. N. & Arlhac, D. Anomalies thermiques dans les eaux du Golfe de Marseille durant l’été 1999. Une explication partielle de la mortalité des invertébrés fixés. *C.R. Acad. Sci., Ser. III* **323**, 853–865 (2000).

83. Vance, T. C. et al. Aquamarine Waters Recorded for the First Time in Eastern Bering Sea. *Earth Space* **10**, 1–16 (1999).

84. Chen, K., Kwon, Y.-O. & Gawarkiewicz, G. Interannual variability of winter-spring temperature in the Middle Atlantic Bight: Relative contributions of atmospheric and oceanic processes. *J. Geophys. Res. Ocean.* 1–14 (2016). doi:10.1002/2015JC011486

85. Florenchie, P. *et al.* Evolution of interannual warm and cold events in the Southeast Atlantic Ocean. *J. Clim.* **17**, 2318–2334 (2004).

86. Mohrholz, V., M., S. & Lutjeharms, J. R. E. The hydrography and dynamics of the Angola-Benguela Frontal Zone and environment in April 1999. *S. Afr. J. Sci.* **97**, 199–208 (2001).

87. Marshall, A. G., Hendon, H. H., Feng, M. & Schiller, A. Initiation and amplification of the Ningaloo Niño. *Clim. Dyn.* **45**, 2367–2385 (2015).

88. Caputi, N., G, J., Peace, A., Jackson, G. & Pearce, A. *The marine heat wave off Western Australia during the summer of 2010/11 – 2 years on*. *Fisheries Research Report [Western Australia]* (2014).

89. Zinke, J. *et al.* Corals record long-term Leeuwin current variability including Ningaloo Niño/Niña since 1795. *Nat. Commun.* **5**, (2014).

90. Lynn, R. J. & Simpson, J. J. The California Current system: The seasonal variability of its physical characteristics. *J. Geophys. Res.* **92**, 12947–12966 (1987).

91. Chelton, D. B., Bratkovich, A. W., Bernstein, R. L. & Kosro, P. M. Poleward flow off central California during the spring and summer of 1981 and 1984. *J. Geophys. Res.* **93**, 10604–10620 (1988).

92. Washburn, L., Fewings, M. R., Melton, C. & Gotschalk, C. The propagating response of coastal circulation due to wind relaxations along the central California coast. *J. Geophys. Res. Ocean.* **116**, (2011).

93. Send, U., Beardsley, R. C. & Winant, C. D. Relaxation from upwelling in the coastal ocean dynamics experiment. *J. Geophys. Res. Ocean.* **92**, 1683–1698 (1987).

94. Qiu, B. Kuroshio and Oyashio currents. in *Ocean Currents: A Derivative of the Encyclopedia of Ocean Sciences* (eds. Steele, J. H., Thorpe, S. A. & Turekian, K. K.) 61–72 (Academic Press, 2001).

95. Yasuda, I. Hydrographic structure and variability in the Kuroshio-Oyashio Transition Area. *J. Oceanogr.* **59**, 389–402 (2003).

96. Nonaka, M. & Xie, S. P. Covariations of sea surface temperature and wind over the Kuroshio and its extension: Evidence for ocean-to-atmosphere feedback. *J. Clim.* **16**, 1404–1413 (2003).

97. Podestá, G. P., Brown, O. B. & Evans, R. H. The annual cycle of satellite-derived sea surface temperature in the southwestern Atlantic ocean. *Journal of Climate* **4**, 457–467 (1991).

98. Severov, D. N., Mordecki, E. & Pshennikov, V. A. SST anomaly variability in Southwestern Atlantic and El Niño/Southern oscillation. *Adv. Sp. Res.* **33**, 343–347 (2004).

99. Putrasahan, D., Kirtman, B. P. & Beal, L. M. Modulation of SST interannual variability in the Agulhas leakage region associated with ENSO. *J. Clim.* **29**, 7089–7102 (2016).

100. de Ruijter, W. P. M. & Boudra, D. B. The wind-driven circulation in the South Atlantic-Indian Ocean—I. Numerical experiments in a one-layer model. *Deep Sea Res. Part A. Oceanogr. Res. Pap.* **32**, 557–574 (1985).

101. Ridgway, K. R. & Dunn, J. R. Mesoscale structure of the mean East Australian Current System and its relationship with topography. *Progress in Oceanography* **56**, 189–222 (2003).

102. Godfrey, J. S., Cresswell, G. R., Golding, T. J., Pearce, A. F. & Boyd, R. The Separation of the East Australian Current. *Journal of Physical Oceanography* **10**, 430–440 (1980).

103. Mata, M. M., Wijffels, S. E., Church, J. A. & Tomczak, M. Eddy shedding and energy conversions in the East Australian Current. *J. Geophys. Res. Ocean.* **111**, (2006).

104. Bowen, M. M., Wilkin, J. L. & Emery, W. J. Variability and forcing of the East Australian Current. *J. Geophys. Res. C Ocean.* **110**, 1–10 (2005).

105. Bull, C. Y. S., Kiss, A. E., Jourdain, N. C., England, M. H. & van Sebille, E. Wind Forced Variability in Eddy Formation, Eddy Shedding, and the Separation of the East Australian Current. *J. Geophys. Res. Ocean.* **122**, 9980–9998 (2017).

106. Sloyan, B. M. & O’Kane, T. J. Drivers of decadal variability in the Tasman Sea. *J. Geophys. Res. Ocean.* **120**, 3193–3210 (2015).

107. Schaeffer, A. & Roughan, M. Subsurface intensification of marine heatwaves off southeastern Australia: The role of stratification and local winds. *Geophys. Res. Lett.* **44**, 5025–5033 (2017).

108. Oliver, E. C. J., O’Kane, T. J. & Holbrook, N. J. Projected changes to Tasman Sea eddies in a future climate. *J. Geophys. Res. Ocean.* **120**, 7150–7165 (2015).

109. Lough, J. M. Climate variability and change on the Great Barrier Reef. in *Oceanographic Processes of Coral Reefs: Physical and Biological Links in the Great Barrier Reef* (ed. Wolanski, E.) 269–300 (CRC Press, 2001).

110. Weller, E., Nunez, M., Meyers, G. & Masiri, I. A climatology of ocean-atmosphere heat flux estimates over the Great Barrier Reef and Coral Sea: Implications for recent mass coral bleaching events. *J. Clim.* **21**, 3853–3871 (2008).

111. Berkelmans, R. Bleaching, upper thermal limits and temperature adaptation in reef corals. *PhD thesis, James Cook Univ.* (2001).

112. Wolanski, E., Andutta, F., Deleersnijder, E., Li, Y. & Thomas, C. J. The Gulf of Carpentaria heated Torres Strait and the Northern Great Barrier Reef during the 2016 mass coral bleaching event. *Estuar. Coast. Shelf Sci.* **194**, 172–181 (2017).

113. Bainbridge, S. J. Temperature and light patterns at four reefs along the Great Barrier Reef during the 2015–2016 austral summer: understanding patterns of observed coral bleaching. *J. Oper. Oceanogr.* **10**, 16–29 (2017).

114. Hughes, T., Zhao, Z., Hintz, R. & Fastook, J. Instability of the Antarctic Ross Sea Embayment as climate warms. *Rev. Geophys.* **55**, 434–469 (2017).

115. Graham, N. A. J. *et al.* Climate warming, marine protected areas and the ocean-scale integrity of coral reef ecosystems. *PLoS One* **3**, e3039 (2008).

116. Ateweberhan, M. & McClanahan, T. R. Relationship between historical sea-surface temperature variability and climate change-induced coral mortality in the western Indian Ocean. *Mar. Pollut. Bull.* **60**, 964–70 (2010).

117. McClanahan, T. R., Ateweberhan, M., Muhando, C. A., Maina, J. & Mohammed, M. S. Effects of climate and seawater temperature vaiation on coral bleaching and mortality. *Ecol. Soc. Am.* **77**, 503–525 (2007).

118. Saji, N. H., Goswami, B. N., Vinayachandran, P. N. & Yamagata, T. A dipole mode in the tropical Indian Ocean. *Nature* **401**, 360–363 (1999).

119. Neelin, J. D. *et al.* ENSO theory. *J. Geophical Res.* **103**, 14261–14290 (1998).

120. Zebiak, S. E. & Cane, M. A. A Model El Nino-Southern Oscillation. *Mon. Weather Rev.* **115**, 2262–2278 (1987).

121. Battisti, D. S. & Hirst, A. C. Interannual Variability in a Tropical Atmosphere-Ocean Model: Influence of the Basic State, Ocean Geometry and Nonlinearity. *J. Atmos. Sci.* **46**, 1687–1712 (1989).

122. Zhang, X. & Mcphaden, M. J. Wind Stress Variations and Interannual Sea Surface Temperature Anomalies in the Eastern Equatorial Pacific. *J. Clim.* **19**, 226–241 (2006).

123. Huang, B., Xue, Y., Wang, H., Wang, W. & Kumar, A. Mixed layer heat budget of the El Niño in NCEP climate forecast system. *Clim. Dyn.* **39**, 365–381 (2012).

124. Glynn, P. W. Widespread Coral Mortality and the 1982–83 El Niño Warming Event. *Environ. Conserv.* **11**, 133–146 (1984).

125. Krishnan, P. *et al.* Elevated sea surface temperature during May 2010 induces mass bleaching of corals in the Andaman. *Curr. Sci.* **100**, 111–117 (2011).

126. Vivekanandan, E., Hussain Ali, M., Jasper, B. & Rajagopalan, M. Thermal thresholds for coral bleaching in the Indian seas. *J. Mar. Biol. Assoc. India* **50**, 209–214 (2008).

127. Sutton, R. T. & Hodson, D. L. R. Atlantic Ocean forcing of North American and European summer climate. *Science* **309**, 115–118 (2005).

128. Diffenbaugh, N. S., Pal, J. S., Giorgi, F. & Gao, X. Heat stress intensification in the Mediterranean climate change hotspot. *Geophys. Res. Lett.* **34**, 1–6 (2007).

129. Di Lorenzo, E. *et al.* Synthesis of Pacific Ocean Climate and Ecosystem Dynamics. *Oceanography* **26**, 68–81 (2013).

130. Stabeno, P. J., Bond, N. A., Kachel, N. B. & Salo, S. A. On the temporal variability of the physical environment over the south-eastern Bering Sea. *Fish. Oceanogr.* **10**, 81–98 (2001).

131. McCabe, R. M. *et al.* An unprecedented coastwide toxic algal bloom linked to anomalous ocean conditions. *Geophys. Res. Lett.* **43**, 10,366-10,376 (2016).

132. Gentemann, C. L., Fewings, M. R. & García-Reyes, M. Satellite sea surface temperatures along the West Coast of the United States during the 2014–2016 northeast Pacific marine heat wave. *Geophys. Res. Lett.* **44**, 312–319 (2017).

133. Mastrandrea, M. D. *et al.* The IPCC AR5 guidance note on consistent treatment of uncertainties: a common approach across the working groups. *Clim. Change* **108**, 675–691 (2011).

**Supplementary Tables**

**Supplementary Table 1 | Characteristic MHW drivers from literature assessment.** Our literature assessment of MHW drivers characterizes the contributions from: large-scale or regional climate modes (e.g. ENSO), atmospheric or oceanic teleconnection processes, and climatological features (e.g. Rossby waves, fronts) [*Mode/Teleconnection* columns]; and local processes (e.g. ocean advection) affecting the MHW heat budget [*Local Process* columns] across four time-scales (synoptic, intraseasonal, interannual and decadal) and classified by typology, i.e. eastern boundary currents (EBC), western boundary currents and extension regions (WBC), tropics, and middle and high latitudes (MHL). For regions where no drivers or processes could be identified from the literature, the box is left blank. For ENSO, PDO, IOD, NAO, and SLP, individual studies may indicate whether MHWs are associated with a positive (+) or negative (-) phase. Case studies with an asterisk (*) have no documented MHWs, but literature identifies processes and modes that cause changes in the mixed layer temperature budget. Numbers correspond to a qualitative confidence assessment for literature documented MHW mode/teleconnection and local processes respectively. Confidence ratings are explained in the main article Methods and Supplementary Information (Supplementary Figure 2) and include very high (_1_), high (_2_), medium (_3_), low (_4_), and very low (_5_) confidence ratings.

|  | | **Timescale** | | | | | | | | |
| --- | --- | --- | --- | --- | --- | --- | --- | --- | --- | --- |
|  | | Synoptic | | Seasonal to Intraseasonal | | Interannual | | Decadal | |  |
| **Typology** | **Case Study** | *Mode/ Teleconnection* | *Local Process* | *Mode/ Teleconnection* | *Local*  *Process* | *Mode/ Teleconnection* | *Local*  *Process* | *Mode/ Teleconnection* | *Local*  *Process* |  |
| EBC | Benguela ^1–3^ |  |  | ABF, RWS, KWO, MJO_1_ | ADV, ASHF_1_ | RWS, KWO_1_ | ADV, VP_1_ |  |  |  |
|  | Leeuwin ^4–7^ |  |  | RASC, SLP(-), LWS_1_ | ADV, EHF, ASHF, VP_1_ | ENSO(-), SLP, RWS_1_ | ADV, ASHF_1_ | PDO(-), ENSO_1_ | ASHF_1_ |  |
|  | California ^8–12^ |  |  | LWS_2_ | ADV, ASHF, VP_1_ | ENSO(+), RWS, SLP(-)_2_ | ASHF, VP, ADV_1_ |  |  |  |
|  | Iberian / Canary ^8,13^ | AB_4_ | ASHF_4_ | NAO(-), RASC, RWS_4_ | ADV, ASHF_4_ | JS_4_ | ASHF_4_ |  |  |  |
|  | Humboldt / Peru^14–18^ |  |  | KWO, RWS_2_ | VP, ADV, ASHF_2_ | ENSO(+), RWS_2_ | ADV, ASHF_2_ |  |  |  |
| WBC | **^*^** Gulf Stream ^19^ | JS_5_ | ASHF, ADV, EHF_5_ | JS, NAO(+)_5_ | ASHF, ADV, EHF. VP_5_ |  |  | AMO_5_ | ADV, EHF_5_ |  |
|  | **^*^** Kuroshio ^20^ | RASC_5_ | ASHF_5_ |  |  | ENSO, RWO_5_ | ADV (fronts), EHF_5_ | PDO, PNA, AL, RWA, RWS_5_ | ASHF_5_ |  |
|  | **^*^** Brazil-Malvinas Confluence ^21–24^ | RASC, RWS, SLP_5_ | ASHF, ADV_5_ | RASC, SLP_5_ | ASHF, ADV, EHF_5_ |  |  |  |  |  |
|  | **^*^** Agulhas ^25,26^ |  |  | RASC, SLP, RWS_5_ | ADV, VP, EHF_5_ | ENSO(-), IOD(-)_5_ | ASHF, ADV, VP_5_ | PDO(-)_5_ | ASHF_5_ |  |
|  | Agulhas Retroflection |  |  | RWS, SLP_5_ | ADV_5_ |  |  |  |  |  |
|  | **^*^** East Australian Current ^27–29^ |  |  | RASC_3_ | ADV, VP, EHF_3_ | ENSO(+), CPEN_3_ | ADV, EHF, VP_3_ | PDO/IPO_3_ | ASHF, ADV, EHF_3_ |  |
|  | East Australian Current Extension^30^ |  |  | BI_5_ | ADV, EHF_5_ | ENSO_3_ | ADV, ASHF_3_ |  |  |  |
| Tropics | Great Barrier Reef  ^31–36^ | AB, RASC, LWS_3_ | ASHF, ADV_3_ |  |  | ENSO(+), CPEN_1_ | VP, ADV, ASHF_1_ | PDO/IPO, RWS_1_ | ASHF, EHF_1_ |  |
|  | Seychelles Is. ^37–40^ |  |  | RASC, SLP, RWS_3_ | ADV, VP_1_ | IOD(+)_2_ | ASHF, ADV, EHF_1_ |  |  |  |
|  | Galápagos Is. ^41,42^ |  |  | RWS_1_ | VP_1_ | ENSO(+), RWS_1_ | VP, ADV, ASHF_1_ | PDO(+)_4_ | ASHF, TD_2_ |  |
|  | Bay of Bengal ^43–45^ | RASC, SLP_4_ | ASHF, TM_4_ | RASC, ASM_4_ | ASHF, ADV_4_ | IOD_4_ | ASHF_4_ |  |  |  |
|  | Caribbean Sea ^46,47^ |  |  |  |  | ENSO(+)_5_ | ASHF_5_ |  |  |  |
| MHL | Mediterranean Sea ^48–54^ | SLP(+), LWS, RWA_1_ | ASHF, VP_1_ |  |  |  |  | AMO_1_ | ASHF_1_ |  |
|  | Bering Sea ^55–61^ | SLP(+)_1_ | ASHF_1_ | AL, LWS_1_ | ASHF, ADV_1_ | ENSO(+), RWA, SLP(+)_1_ | ASHF_1_ | AL, PDO_1_ | ASHF_1_ |  |
|  | Northwest Atlantic ^62–67^ |  |  | JS, RASC, RWS, SLP_1_ | ASHF_1_ |  |  |  |  |  |
|  | Northeast Pacific ^67–72^ | SLP(+), LWS_1_ | ASHF, EHF_1_ | AL_1_ | ASHF_1_ | ENSO(+), RWA_1_ | AL, SLP(+)_1_ | NPO(+), RWS, NPGO(+)_1_ | SLP(+)_1_ |  |
|  | South Central Pacific^73^ |  |  |  |  | ENSO(+), RWA_5_ | ASHF, ADV_5_ |  |  |  |

| **Large-scale and regional climate modes** | | **Teleconnection processes & climatological features** | | **Local processes affecting the mixed layer temperature budget** | |
| --- | --- | --- | --- | --- | --- |
| ENSO(+/-)  CPEN  IPO  PDO(+/-)  IOD(+/-)  MJO  NAM  NAO(+/-)  NPGO(+/-)  NPO  AMO  SAM  ASM | El Niño-Southern Oscillation  Central Pacific El Niño  Interdecadal Pacific Oscillation  Pacific Decadal Oscillation  Indian Ocean Dipole  Madden-Julian Oscillation  Northern Annular Mode  North Atlantic Oscillation  North Pacific Gyre Oscillation  North Pacific Oscillation  Atlantic Multidecadal Oscillation  Southern Annular Mode  Asian Summer Monsoon | AB  AL  SLP(+/-)  JS  PNA  RWA  ABF  BI  KWO  RWO  RWS  RASC  LWS | Atmospheric Blocking  Aleutian Low  Sea Level Pressure  Jet Stream position  Pacific North American Pattern  Rossby Wave (Atmospheric)  Angola-Benguela Front  Baroclinic Instability  Kelvin Wave (Oceanic)  Rossby Wave (Oceanic)  Regional wind stress change  Regional air-sea coupling  Local wind stress change | ADV  EHF  ASHF  VP | Ocean Advection  Eddy heat flux  Air-sea heat flux  Vertical Processes (entrainment, turbulent mixing, thermocline deepening) |

**Supplementary Table 2 | Characteristics of strong marine heatwaves identified in the literature (1982-2016).** MHWs were identified using the quantitative definition^74^ applied here to derive metrics (but based on a 98^th^-percentile threshold). [Note: Those MHWs identified in the literature that have a dash ‘-’ did not meet the 98^th^ percentile criteria although identified in the literature with the corresponding references. Further, some of the closely separated MHW events defined here (e.g. Humboldt/Peru Current region in 1982, 1983 and 1997/98; and the northeast Pacific region in 2013/14 and 2014/15) would be considered a continuous MHW if a weaker threshold was used].

|  |  |  | | **Metrics** | | | |
| --- | --- | --- | --- | --- | --- | --- | --- |
| **Typology** | **Case Study** | **Marine Heatwaves (Ref #)** | **Start Date (>98%)** | | **End Date**  **(<98%)** | **Max Intensity**  **[ºC (Date)]** | **Max Area**  **[Mkm^2^ (Date)]** |
| EBC | Benguela | 1982/83^3^  1984^3,71^  1995^2^  2001^1^ | 21/12/1982  8/3/1984  5/2/1995  21/3/1995  11/4/1995  27/4/2001 | | 31/1/1983  12/3/1984  19/2/1995  1/4/1995  21/4/1995  20/5/2001 | 4.8 (22/1/1983)  4.6(9/3/1984)  4.7(7/2/1995)  5.7(22/3/1995)  6.7(16/4/1995)  5.3 (14/5/2001) | 1.4 (24/1/1983)  0.13(9/3/1984)  0.2 (17/2/1995)  0.3 (27/3/1995)  0.6 (17/4/1995)  0.7 (16/5/2001) |
|  | Leeuwin | 1989 ^75^  1999/2000 ^75,76^  2011^4–7^  2012^75^  2012/13^75^ | -  -  8/2/2011  20/1/12  2/1/2013 | | -  -  23/3/2011  29/1/12  10/1/2013 | -  -  6.8(26/2/2011)  5.1(28/1/11)  4.0 (2/1/2013) | -  -  0.95(4/3/2011)  0.3 (17/1/11)  0.24 (5/1/2013) |
|  | Baja California | 1982/83^8,9,12^  1997^10,12^  2015/16^72^ | 10/2/1983  14/11/1997  30/12/1997  10/2/1998  1/7/2015 | | 5/3/1984  11/12/1997  14/1/1998  20/2/1998  2/1/2016 | 4.0(23/2/1983)  4.6(26/11/1997)  4.2(31/12/1997)  3.5(19/2/1998)  6.4(12/9/2015) | 0.12(24/2/1983)  0.37(6/12/1997)  0.39 (3/1/1998)  0.17(16/2/1998)  21.2(2/11/2015) |
|  | Iberian / Canary | - | - | | - | - | - |
|  | Humboldt / Peru | 1982^14,16^  1983^15^  1997/98^17,18^ | 23/8/1982  14/10/1982  26/11/1982  5/1/1983  30/5/1997  9/7/1997  3/9/1997  24/9/1997  25/11/1997 | | 7/10/1982  18/11/1982  14/12/1982  24/7/1983  18/6/1997  25/8/1997  17/9/1997  17/11/1997  28/12/1997 | 4.2(10/9/1982)  3.9(16/11/1982)  5.5(11/12/1982)  9.6 (6/5/1983)  4.1(2/6/1997)  6.2(21/7/1997)  6.5(7/9/1997)  6.2(7/11/1997)  6.9(28/11/1997) | 2.1(29/9/1982)  0.25(16/11/1982)  1.0(9/12/1982)  4.4 (24/6/1983)  5.4(12/6/1997)  4.7(18/8/1997)  8.1 (4/9/1997)  7.0(26/9/1997)  4.1(5/12/1997) |
| WBC | East Australian Current Extension | 2016^30^ | 7/12/2015 | | 12/4/2016 | 7.5(8.2.2016) | 0.7(6/3/2016) |
|  | Agulhas Retroflection | 1985^77^ | 29/11/1985 | | 5/12/1985 | 0.2(2/12/1985) | 5.7(29/11/1985) |
| Tropics | Great Barrier Reef / Coral Sea | 1998^18,31,33,36,40^  2001/02 ^31,36^  2015/16 ^78–81^ | 9/2/1998  30/12/2001  28/2/2016 | | 18/3/1998  15/1/2002  4/4/2016 | 3.4(4/3/1998)  3.3 (6/1/2002)  4.0 (15/3/2016) | 0.14 (5/3/1998)  0.5(8/1/2002)  2.6 (12/3/2016) |
|  | Seychelles Is. | 1997/98^37,39,40^ | 14/1/1998  14/2/1998 | | 3/2/1998  22/2/1998 | 3.7(25/1/1998)  3.4(21/2/1998) | 0.4(18/1/1998)  0.9(18/2/1998) |
|  | Galápagos Is. (Eq. Pac) | 1982/83^41^  1987^41^  1992^41^  1997/98^42^ | 5/5/1983  -  -  14/7/1997 | | 31/7/1983  3/6/1998 | 9.56(6/5/1983)  6.9(28/11/1997) | 4.7(5/6/1983)  11(9/11/1997) |
|  | Bay of Bengal | 2010^44^ | 4/5/2010 | | 6/6/2010 | 3.4(15/5/2010) | 1.1(14/5/2010) |
|  | Caribbean Sea | 1982/83^46^  1987/88^47^  1989/90^47^ | -  -  - | | -  -  - | -  -  - | -  -  - |
| MHL | Mediterranean Sea | 1999 ^82^  2003^48,50–52,54^ | -  6/9/2003  8/8/2003 | | -  16/7/2003  4/9/2003 | -  5.5(14/6/2003)  4.6 (29/8/2003) | -  0.5(16/6/2003)  1.2(23/8/2003) |
|  | Bering Sea | 1997^55–58,61,83^ | 31/5/1997 | | 27/6/1997 | 5.1(12/6/1997) | 0.05(4/6/1997) |
|  | Northwest Atlantic Ocean | 2012 ^64,67,84^ | 18/3/2012  17/4/2012  19/5/2012  21/6/2012  19/9/2012  31/10/2012 | | 25/3/2012  28/4/12  31/5/2012  10/9/2012  13/10/2012  27/11/2012 | 10.3 (23/3/2012)  5.4(17/4/2012)  7.5(23/5/2012)  9.2(6.7/2012)  6.1 (8/10/2012)  8.1(27/11/2012) | 0.3(22/3/2012)  0.3(25/4/2012)  0.2(26/5/2012)  0.1(1/8/2012)  0.3(8/10/12)  0.2(3/11/2012) |
|  | Northeast Pacific Ocean | 2013/14 ^68,69,71,72^ | 14/11/2013  28/7/2014 | | 30/6/2014  31/8/2015 | 4.8(3/6/2014)  6.7(28/6/2015) | 4.5(17/1/2014)  11.7(15/2/2015) |
|  | South Central Pacific Ocean | 2009/10 ^73^ | 9/11/2009 | | 2/3/2010 | 6.0(24//12/2009) | 3.9(25/12/2009) |

**Supplementary Table 3 | Literature review criteria for marine heatwave case studies.** This is informed by the qualitative marine heatwave definition^74^ focused during the period of improved data availability and coverage beginning in 1950.

| **Marine Heatwave Literature Review Criteria** |
| --- |
| 1. Expressed or measured through sea surface temperature |
| 1. Discrete event with a fixed start and end period |
| 1. Having an extended spatial area |
| 1. Exceeding climatological norms |
| 1. Occurring during or after 1950 |

**Supplementary Table 4** | Benguela Current region MHW event case studies

| **Date** | **Location** | **Reference** |
| --- | --- | --- |
| Austral Summer 1982/83 | Benguela Current | ^3^ |
| Mar-Apr 1984 | Eastern South Atlantic | ^85^ |
| Late 1985/86 | Southern Benguela Current region | ^77^ |
| Feb-Mar 1995 | Benguela Current | ^2^ |
| Mar-Apr 1995 | Eastern South Atlantic | ^85^ |
| Feb-Apr, 2001 | Benguela Current | ^1^ |

**Supplementary Table 5 |** Leeuwin Current region MHW event case studies

| **Date** | **Location** | **Reference** |
| --- | --- | --- |
| 1955/56, 1960/61, 1961/62, 1962/63,1966/67, 1973/74,  1982/83, 1996/97, 2010/11 | Ningaloo Reef to Cape Leeuwin | ^76^ |
| 1999/2000 | Ningaloo Reef to Cape Leeuwin | ^75,76^ |
| 2012  2012/13 | Ningaloo Reef to Cape Leeuwin | ^75^ |
| Feb-Mar 2011 | Ningaloo Reef to Cape Leeuwin | ^5–7,75^ |

**Supplementary Table 6 |** California Current region MHW event case studies

| **Date** | **Location** | **Reference** |
| --- | --- | --- |
| 1957/58 | Scripps Pier | ^8^ |
| 1972/73 | Scripps Pier | ^8^ |
| 1976/77 | Scripps Pier | ^8^ |
| 1982/83 | Scripps Pier | ^8,9^ |
| Feb-July 1984 | Point Conception to San Francisco | ^91^ |
| Oct 1997- Jan 1998 | North Pacific Subtropical Gyre east of 125°W | ^10^ |
| 2015/16 | Off Baja California | ^72^ |

**Supplementary Table 7 |** Iberian/Canary region warm SST event case studies.

| **Date** | **Location** | **Reference** |
| --- | --- | --- |
| 1978, 1979/80, 1981/82, 1983/84 | Canary Current | ^8^ |

**Supplementary Table 8 |** Humboldt/Peru region warm SST event case studies.

| **Date** | **Location** | **Reference** |
| --- | --- | --- |
| 1976 | Peruvian coast (~10^o^S) | ^16^ |
| 1982/83 late summer | Peruvian coast | ^14–16^ |
| 1997/98 | Peruvian coast (San Juan, 15^o^S) | ^17,18^ |

**Supplementary Table 9 |** Agulhas Retroflection region MHW event case studies

| **Date** | **Location** | **Reference** |
| --- | --- | --- |
| 1985/86 | Agulhas Retroflection/SE Atlantic | ^77^ |

**Supplementary Table 10 |** Tasman Sea region MHW event case studies

| **Date** | **Location** | **Reference** |
| --- | --- | --- |
| Sep 2015-May 2016 | West Tasman Sea | ^30^ |

**Supplementary Table 11 |** Great Barrier Reef region MHW event case studies

| **Date** | **Location** | **Reference** |
| --- | --- | --- |
| 1997-1998 | Great Barrier Reef | ^35,109^ |
| 2002 | Great Barrier Reef | ^31,110,111^ |
| 2016 | Great Barrier Reef | ^80,81,112–114^ |

**Supplementary Table 12 |** Eastern Africa/Seychelles Islands region MHW event case studies

| **Date** | **Location** | **Reference** |
| --- | --- | --- |
| 1998 | Seychelles, Western Indian Ocean | ^39,115,116^ |

**Supplementary Table 13 |** EEP/Galapagos Islands region MHW event case studies

| **Date** | **Location** | **Reference** |
| --- | --- | --- |
| 1982/83 | Galapagos/EEP | ^41,46,124^ |
| 1987 | Galapagos/EEP | ^41^ |
| 1992 | Galapagos/EEP | ^41^ |

**Supplementary Table 14 |** Bay of Bengal region MHW event case studies

| **Date** | **Location** | **Reference** |
| --- | --- | --- |
| 1998 | Bay of Bengal | ^43^ |
| 1998, 2002, 2005,  2010 | Bay of Bengal | ^125^ |

**Supplementary Table 15 |** Caribbean Sea region MHW event case studies

| **Date** | **Location** | **Reference** |
| --- | --- | --- |
| 1982/83 | Caribbean Sea | ^46^ |
| 1987/88, 1989/90 | Jamaica | ^47^ |

**Supplementary Table 16 |** Mediterranean Sea region MHW event case studies

| **Date** | **Location** | **Reference** |
| --- | --- | --- |
| Summer 1999 | Mediterranean Sea | ^82^ |
| Apr-Aug 2003 | Mediterranean Sea (Tyrrhenian) | ^49,51,52,54^ |

**Supplementary Table 17 |** Bering Sea region MHW event case studies

| **Date** | **Location** | **Reference** |
| --- | --- | --- |
| June 1997 | Eastern shelf Bering Sea | ^57–59,61,130^ |
| 2000-2005 | Eastern Bering Sea | ^58^ |

**Supplementary Table 18 |** Northwest Atlantic Ocean region MHW event case studies

| **Date** | **Location** | **Reference** |
| --- | --- | --- |
| Mar-Aug 2012 | Northwest Atlantic | ^63–65^ |

**Supplementary Table 19 |** Northeast Pacific Ocean region MHW event case studies

| **Date** | **Location** | **Reference** |
| --- | --- | --- |
| Dec 2013-2015 | 41-45ºN, 145-150ºW | ^68–72^ |

**Supplementary Table 20 |** South Pacific Ocean region MHW event case studies

| **Date** | **Location** | **Reference** |
| --- | --- | --- |
| Sep-Dec 2009 | South-Central Pacific | ^73^ |

**Supplementary Figures**

**Supplementary Figure 1 | Local processes can alter surface mixed layer temperatures.**

We define ‘processes’ to be the local factors that affect the evolution of ocean temperature within the surface mixed layer at a certain location, i.e. net surface heat flux (comprising of net shortwave radiation flux + longwave radiation flux + sensible heat flux + latent heat flux at the ocean surface), radiative heat loss at the base of the mixed layer, horizontal advection (from the mean circulation or high-frequency small-scale flow), vertical entrainment, and mixing.


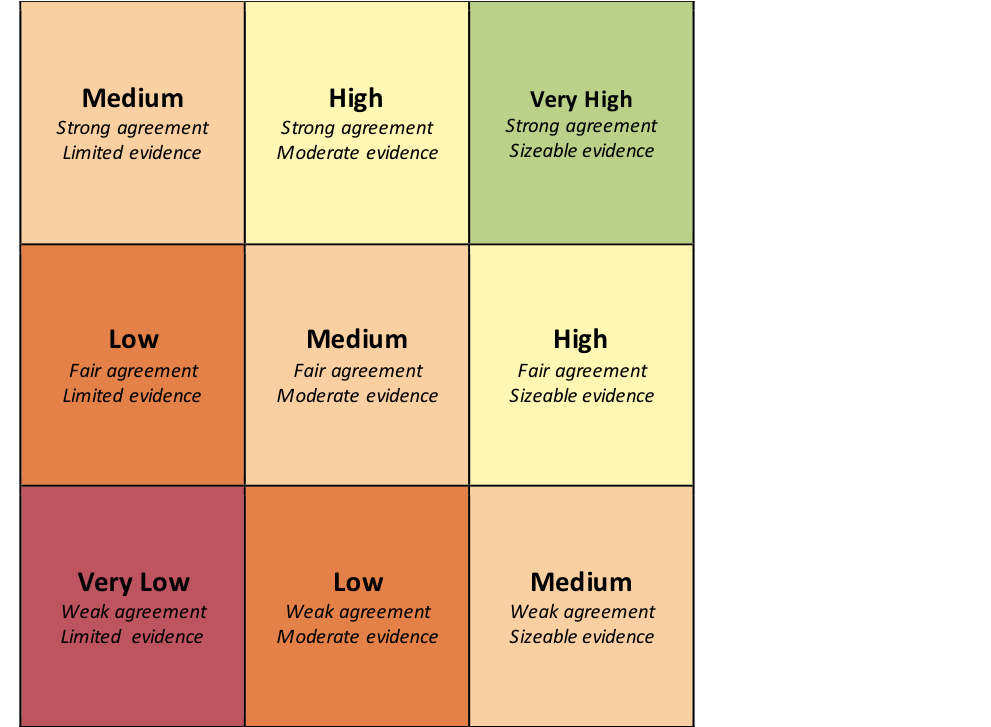


**Supplementary Figure 2 | Qualitative confidence matrix.** The amount of evidence available and the levels of agreement in the evidence determine the confidence rating (Modified from Mastrandrea et al.^133^ ). In the present study, our confidence rating in characterizing the MHW mechanisms for a particular case study region is very high when our literature synthesis includes multiple published studies of identified MHW events in the region with consistent causative mechanisms (i.e. there is sizeable evidence and strong agreement around the key drivers of MHWs in the region).


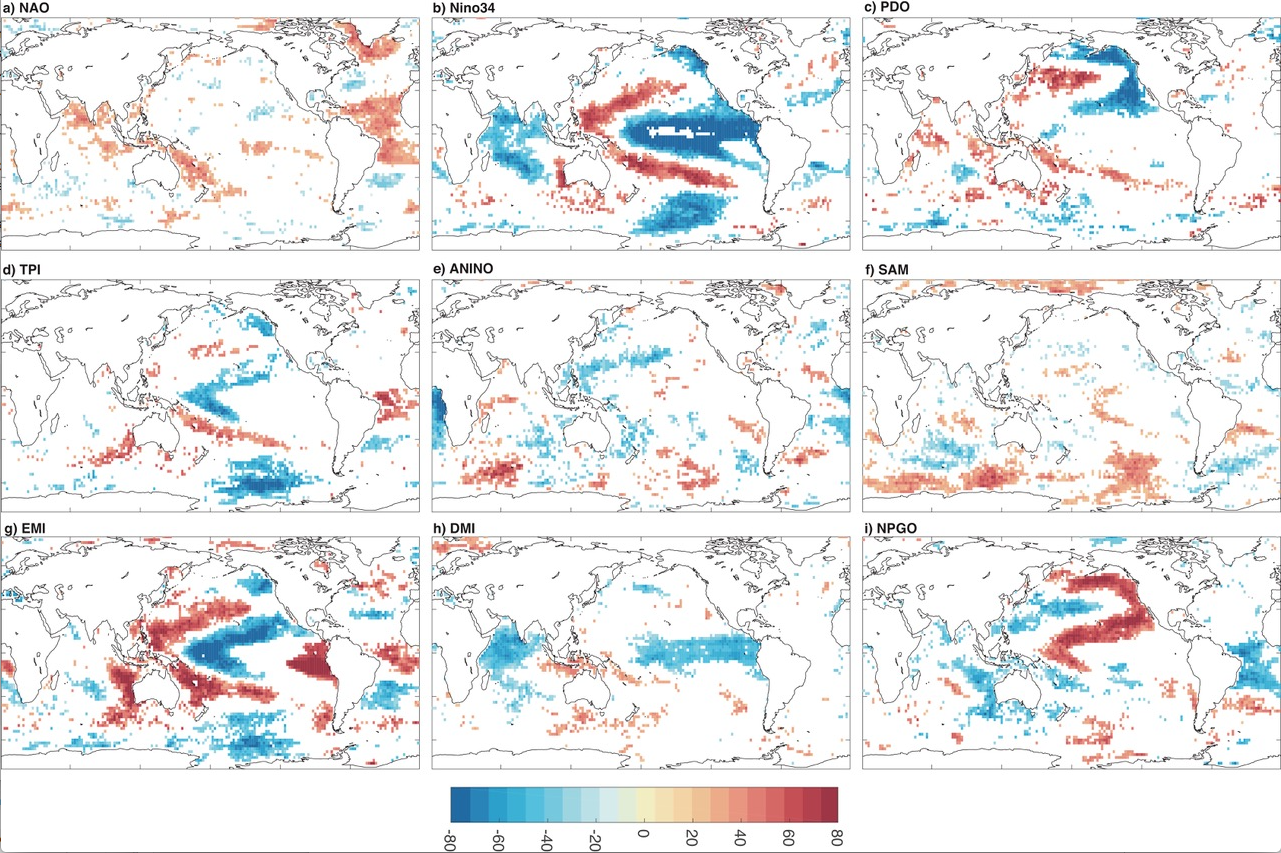


**Supplementary Figure 3 | Percentage change in MHW days in mode negative phase.** Percentage of days in which MHWs increase or decrease during the negative phase of each climate mode.


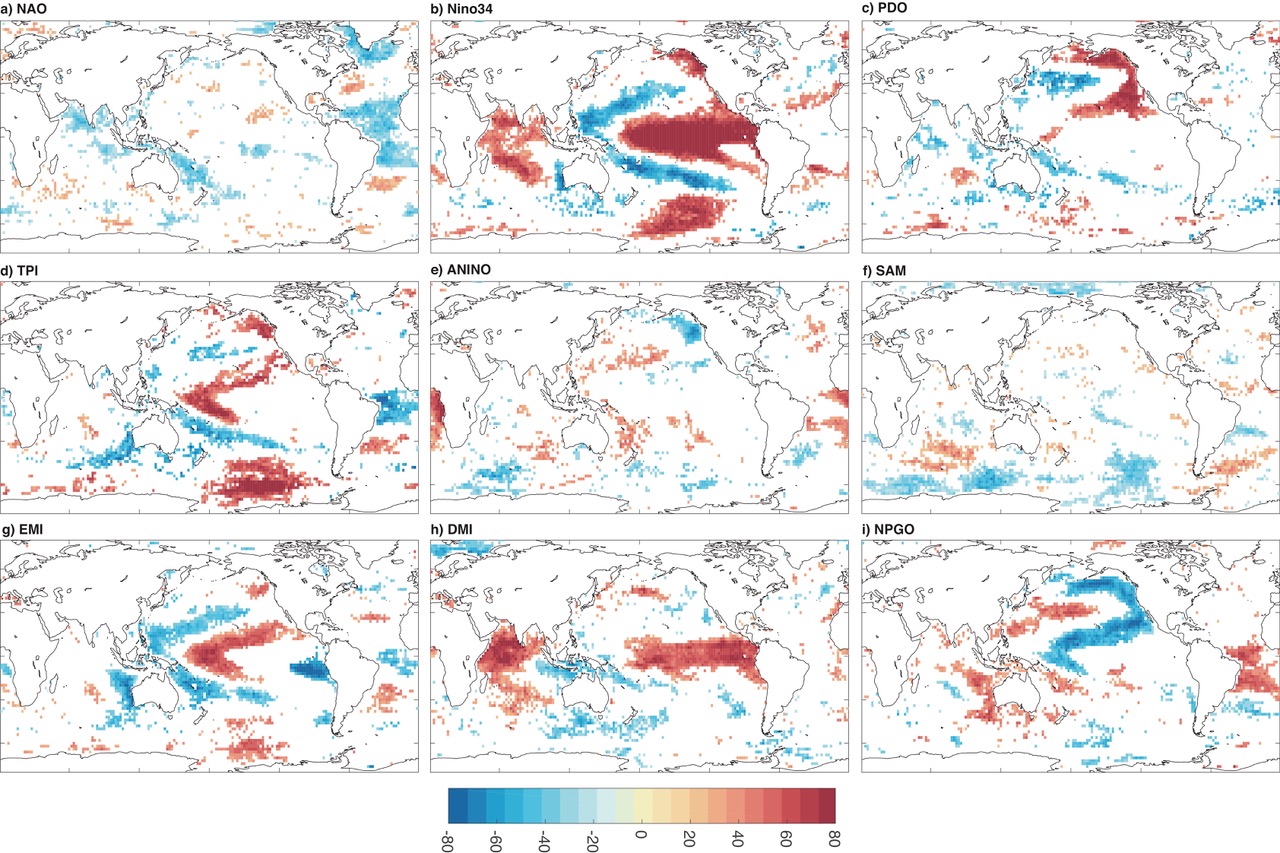


**Supplementary Figure 4 | Percentage change in MHW days in mode positive phase.** Percentage of days in which MHWs increase or decrease during the positive phase of each climate mode.


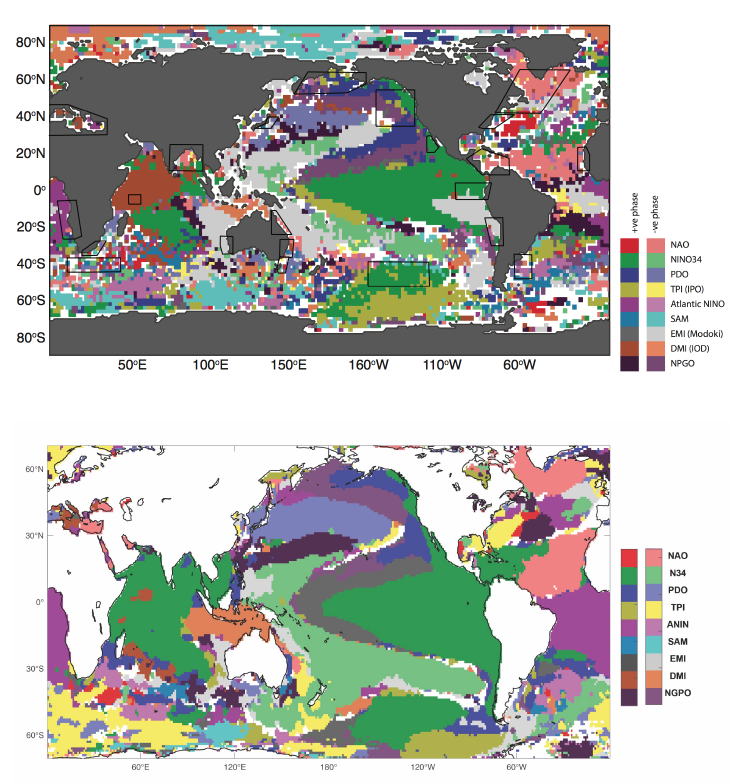


**Supplementary Figure 5 | MHW days and SSTA increases in relation to climate mode.** Top: Mode/phase associated with the greatest enhancement of MHW days (see Fig. 3b in the manuscript) from a composite analysis. Bottom: Mode phase associated with the greatest SST anomaly increase (determined via regression of SST anomalies onto the normalized mode time series). There are regional differences between these panels.


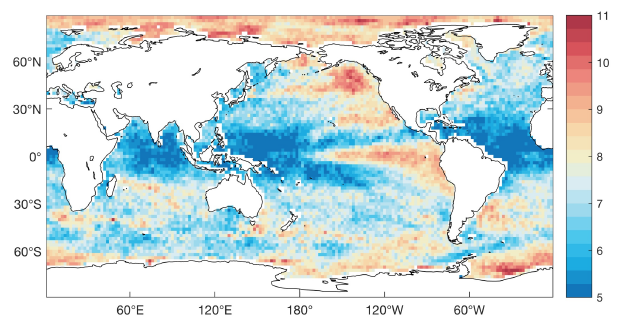


**Supplementary Figure 6 | Median percentage of MHW days per year.** Note: Despite the definition for MHW exceeding the 90^th^ percentile, the median values are typically <10% at any location because of the 5-day criterion for MHW occurrence. The global median value is ~7.5%. The enhancement and suppression of MHW days in the figures that follow (Figures S7-S15) are relative to the values shown in this figure.


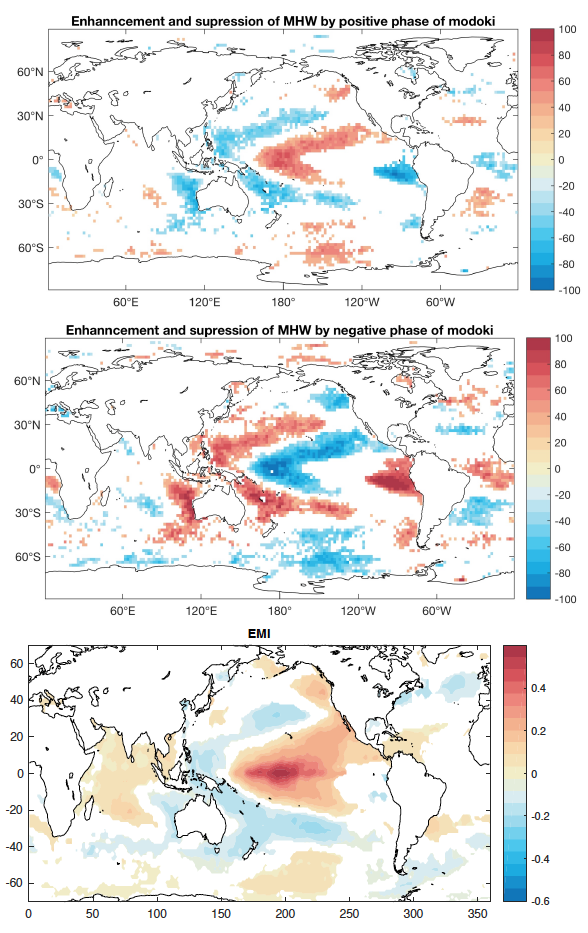


**Supplementary Figure 7 | Percentage change in MHW days according to EMI phase.** Percentage increase or decrease in MHW days for the (top) positive, and (middle) negative phase of the EMI (where the increase or decrease is significant at the 10% level based on a Monte Carlo simulation). (Bottom) The associated SST anomaly regression (where *p*<0.1).


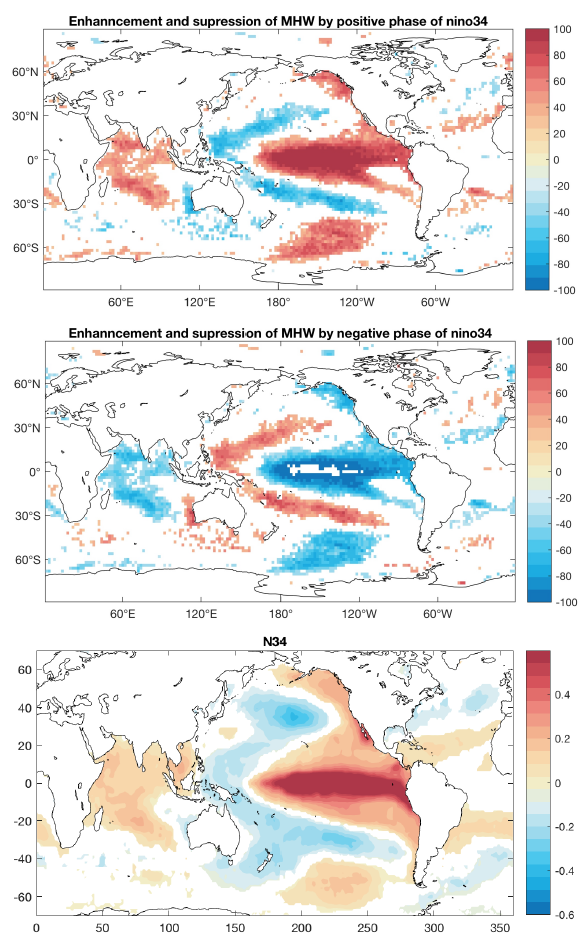


**Supplementary Figure 8 | Percentage change in MHW days according to Ni**ñ**o34 phase.** Percentage increase or decrease in MHW days for the (top) positive, and (middle) negative phase of the Niño34 (where the increase or decrease is significant at the 10% level based on a Monte Carlo simulation). (Bottom) The associated SST anomaly regression (where *p*<0.1).


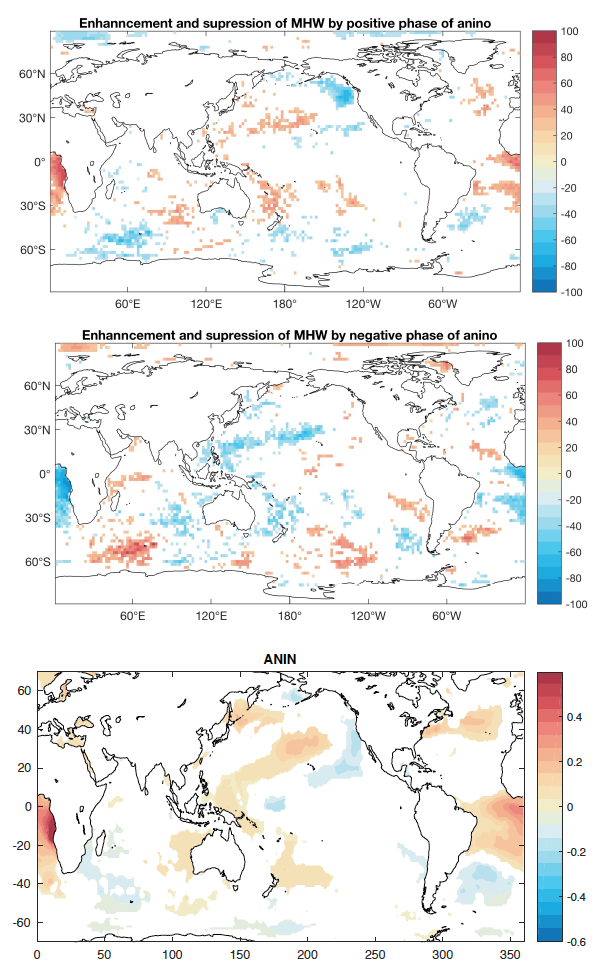


**Supplementary Figure 9 | Percentage change in MHW days according to ANINO phase.** Percentage increase or decrease in MHW days for the (top) positive, and (middle) negative phase of the Atlantic Niño (ANINO/ ATLN1) (where the increase or decrease is significant at the 10% level based on a Monte Carlo simulation). (Bottom) The associated SST anomaly regression (where *p*<0.1).


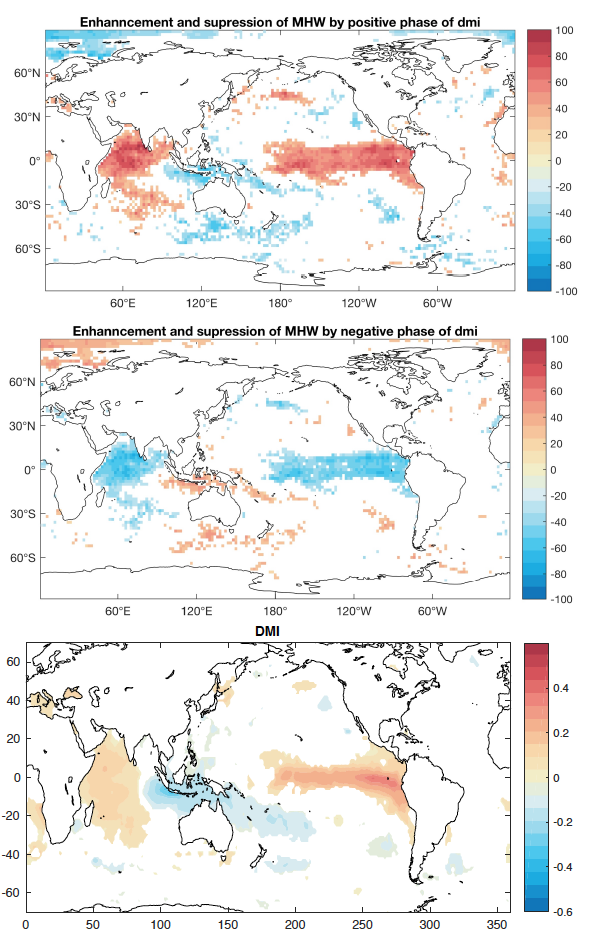


**Supplementary Figure 10 | Percentage change in MHW days according to DMI phase.** Percentage increase or decrease in MHW days for the (top) positive, and (middle) negative phase of the DMI (where the increase or decrease is significant at the 10% level based on a Monte Carlo simulation). (Bottom) The associated SST anomaly regression (where *p*<0.1).


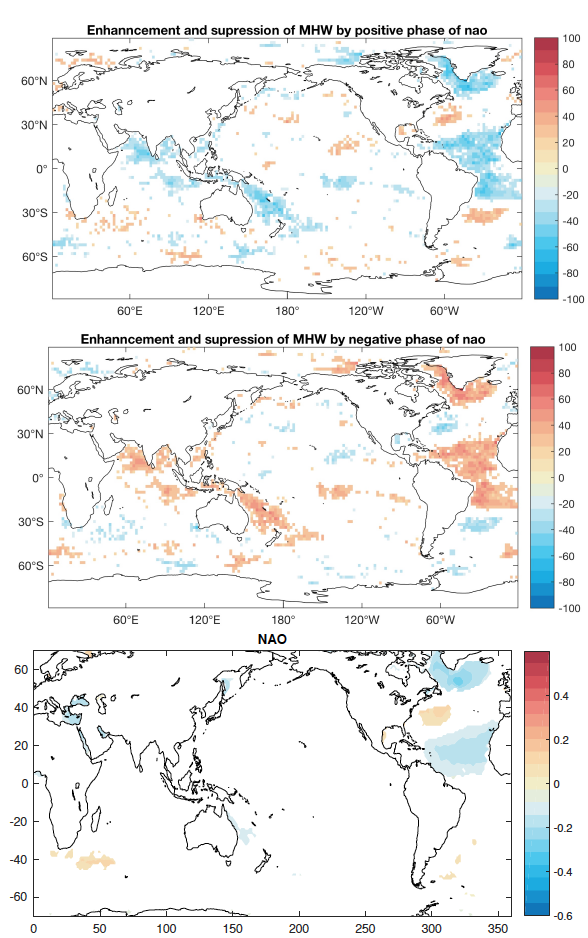


**Supplementary Figure 11 | Percentage change in MHW days according to NAO phase.** Percentage increase or decrease in MHW days for the (top) positive, and (middle) negative phase of the NAO (where the increase or decrease is significant at the 10% level based on a Monte Carlo simulation). (Bottom) The associated SST anomaly regression (where *p*<0.1).


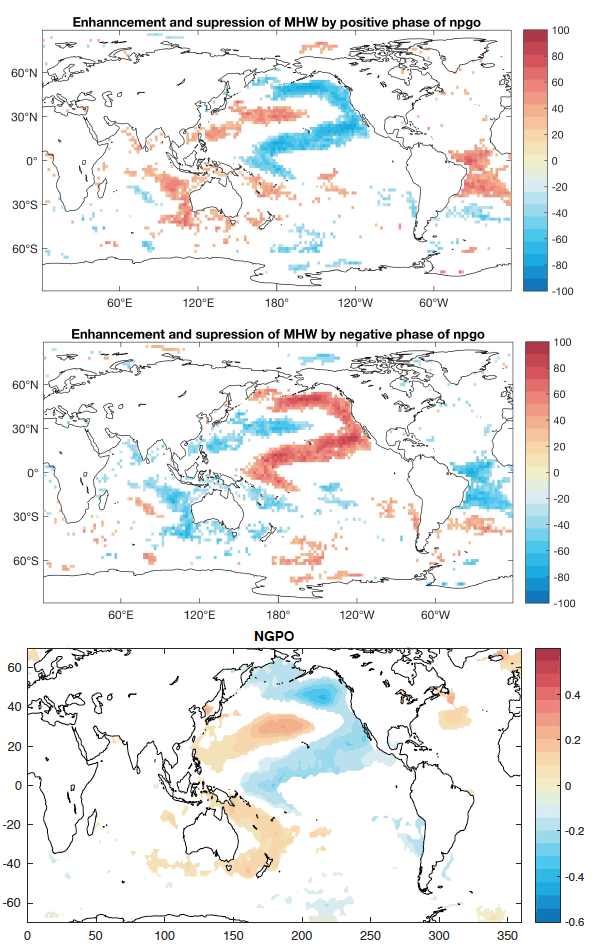


**Supplementary Figure 12 | Percentage change in MHW days according to NPGO phase.** Percentage increase or decrease in MHW days for the (top) positive, and (middle) negative phase of the NPGO (where the increase or decrease is significant at the 10% level based on a Monte Carlo simulation). (Bottom) The associated SST anomaly regression (where *p*<0.1).


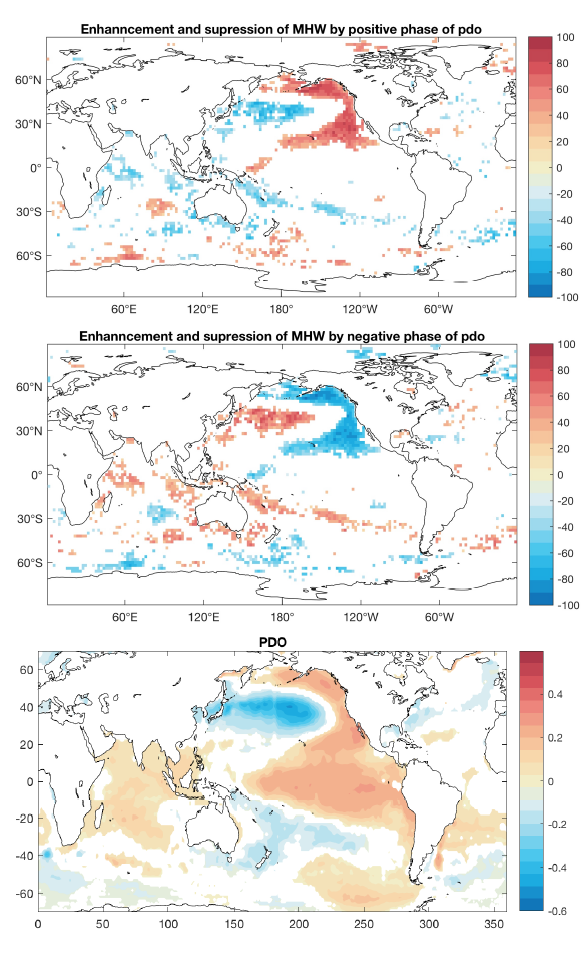


**Supplementary Figure 13 | Percentage change in MHW days according to PDO phase.** Percentage increase or decrease in MHW days for the (top) positive, and (middle) negative phase of the PDO (where the increase or decrease is significant at the 10% level based on a Monte Carlo simulation). (Bottom) The associated SST anomaly regression (where *p*<0.1).


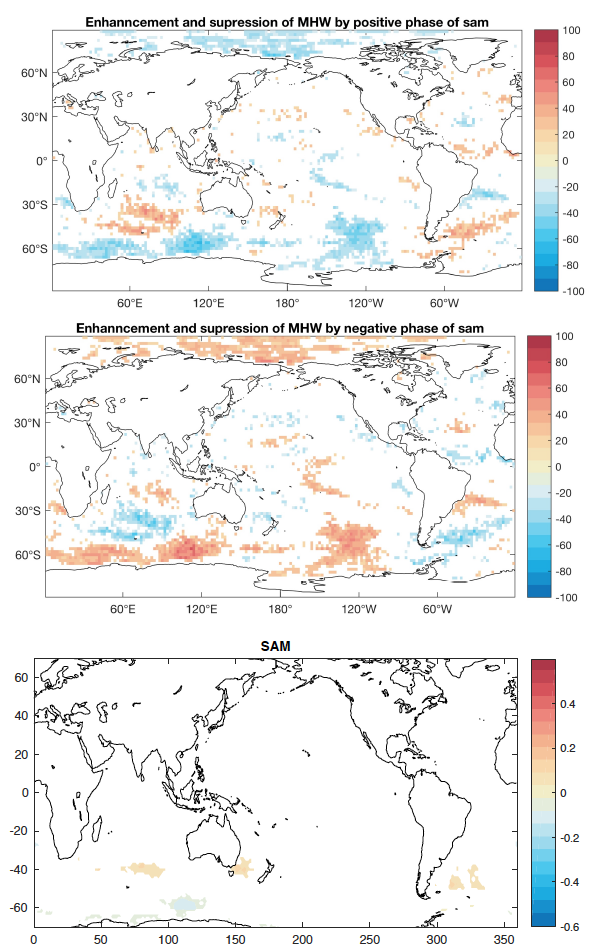


**Supplementary Figure 14 | Percentage change in MHW days according to SAM phase.** Percentage increase or decrease in MHW days for the (top) positive, and (middle) negative phase of the SAM (where the increase or decrease is significant at the 10% level based on a Monte Carlo simulation). (Bottom) The associated SST anomaly regression (where *p*<0.1).


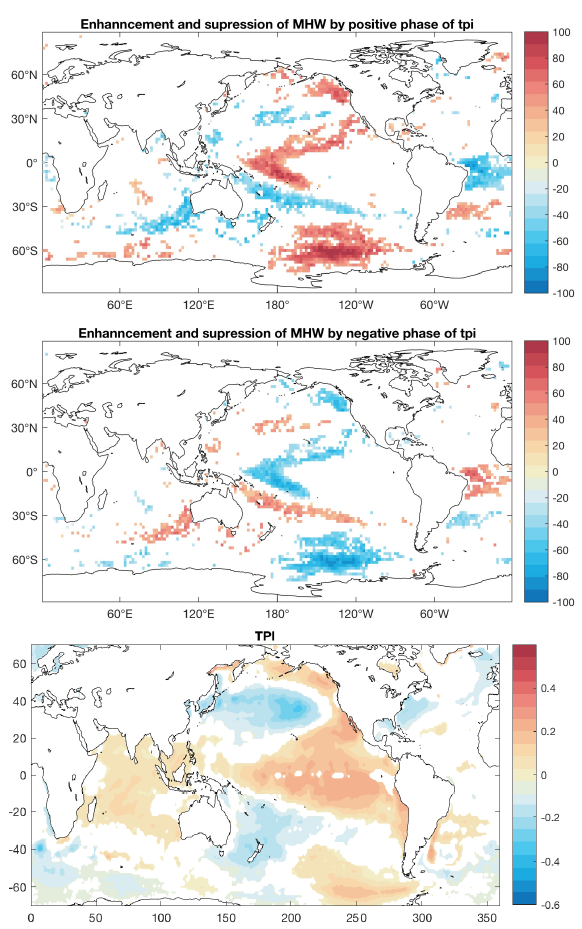


**Supplementary Figure 15 | Percentage change in MHW days according to TPI phase.** Percentage increase or decrease in MHW days for the (top) positive, and (middle) negative phase of the TPI (where the increase or decrease is significant at the 10% level based on a Monte Carlo simulation). (Bottom) The associated SST anomaly regression (where *p*<0.1).
